# Occupancy‐derived thermal affinities reflect known physiological thermal limits of marine species

**DOI:** 10.1002/ece3.6407

**Published:** 2020-06-15

**Authors:** Thomas J. Webb, Aaron Lines, Leigh M. Howarth

**Affiliations:** ^1^ Department of Animal and Plant Sciences University of Sheffield Sheffield UK; ^2^ Life Sciences Centre Dalhousie University Halifax NS Canada

**Keywords:** biodiversity informatics, climate change, critical temperature, gridded global sea temperature, OBIS, open biodiversity data, thermal safety margin, thermal tolerance

## Abstract

Predicting how species will respond to increased environmental temperatures is key to understanding the ecological consequences of global change. The physiological tolerances of a species define its thermal limits, while its thermal affinity is a summary of the environmental temperatures at the localities at which it actually occurs. Experimentally derived thermal limits are known to be related to observed latitudinal ranges in marine species, but accurate range maps from which to derive latitudinal ranges are lacking for many marine species. An alternative approach is to combine widely available data on global occurrences with gridded global temperature datasets to derive measures of species‐level “thermal affinity”—that is, measures of the central tendency, variation, and upper and lower bounds of the environmental temperatures at the locations at which a species has been recorded to occur. Here, we test the extent to which such occupancy‐derived measures of thermal affinity are related to the known thermal limits of marine species using data on 533 marine species from 24 taxonomic classes and with experimentally derived critical upper temperatures spanning 2–44.5°C. We show that thermal affinity estimates are consistently and positively related to the physiological tolerances of marine species, despite gaps and biases in the source data. Our method allows thermal affinity measures to be rapidly and repeatably estimated for many thousands more marine species, substantially expanding the potential to assess vulnerability of marine communities to warming seas.

## Graphical abstract

We develop a workflow for matching marine species occurrence records to global sea temperature datasets, to rapidly estimate species‐level thermal affinities. We show that thermal affinities estimated in this way are closely correlated with critical thermal limits derived from experiments for 533 marine species from 24 taxonomic classes. Our method allows thermal affinity measures to be rapidly and repeatably estimated for many thousands more marine species, vastly expanding the potential to assess vulnerability of marine communities to warming seas.

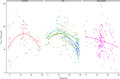

## 1 INTRODUCTION

Predicting how individual species will respond to increased environmental temperatures is key to understanding the wider ecological consequences of global change. Temperature influences the physiological and behavioral responses of individuals (Sunday, Bates, & Dulvy, 2011), and as environmental temperatures move further from a species' optimal temperature, the ability of the individual to grow and reproduce is reduced (Pörtner & Farrell, 2008). This has direct consequences for the abundance and distributions of species in all environments (Day, Smith, Edgar, & Bates, 2018; Sunday et al., 2011; Waldock, Smith, Edgar, Bird, & Bates, 2019), and in marine systems, distributional shifts have tracked local changes in climate (Pinsky, Selden, & Kitchel, 2020; Pinsky, Worm, Fogarty, Sarmiento, & Levin, 2013). Documenting the thermal tolerances of a wide range of species is thus an important step toward forecasting future distributional shifts (Sunday, Bates, & Dulvy, 2012) and community changes.

Experimental derivations of thermal tolerance limits have long been the gold standard for understanding the thermal biology of species and include methods based on both lethal and critical limits. For instance, estimating lethal temperatures (typically the temperature lethal to 50% of a population) has a long history of use in studies of a wide range of taxa (e.g., Fry, 1967; Lutterschmidt & Hutchison, 2011). However, because this technique is logistically difficult and can be ethically problematic (Eme & Bennett, 2009), the critical thermal methodology was developed and adopted as the preferred method for experimental derivations of thermal tolerance (Lutterschmidt & Hutchison, 2011). Critical thermal tests, including both critical thermal minimum (CT_min_) and critical thermal maximum (CT_max_), are preferred because they use relatively fewer individuals and provide a rapid, nonlethal assessment by measuring the loss of key ecological functions rather than mortality. For example, critical temperatures can be marked by the thermal point at which locomotion is lost or by the onset of muscular spasms (Beitinger, Bennett, & McCauley, 2000; Lutterschmidt & Hutchison, 2011). However, use of these physiological thermal traits to assess climatic vulnerability is limited (Pacifici et al., 2015), because experimental derivations of thermal tolerances remain hard to obtain, and those studies that do exist are not standardized with respect to heating rate and temperature acclimatization protocols (Comte & Olden, 2017; Lutterschmidt & Hutchison, 2011). Nonetheless, their importance is reflected in recent efforts to compile global databases of experimentally derived thermal limits for marine and freshwater fish (Comte & Olden, 2017) and for a wide range of species across all environments (Bennett et al., 2018a,b).While experimentally derived thermal tolerances have remained difficult to obtain, the literature on “realized thermal niches” of species has expanded. The thermal tolerance limits derived experimentally represent the fundamental thermal niche of the species—the temperature range at which the species *could* survive, in the absence of predation, competition, or habitat heterogeneity. The realized thermal niche of a species represents the environmental temperatures at which individuals are *actually* observed to occur (Magnuson, Crowder, & Medvick, 1979). In marine species especially, there is a good correspondence between these two measures, with latitude and experienced temperature extremes proving a good predictor of thermal limits (Sunday et al., 2011, 2019). This has led to a decade of work using various estimates of thermal limits and environmental temperature to predict how climate change will drive changes in marine species and communities (see Pinsky et al., 2020 for recent review). Indices based on the environmental temperatures at which species occur have also been developed. For instance, the Species Temperature Index of a species, calculated as the mid‐point of the realized thermal niche (Devictor et al., 2012), has been shown to relate to the environmental temperature at which the maximum local abundance of a species is attained (Stuart‐Smith, Edgar, Barrett, Kininmonth, & Bates, 2015). This has been extended to whole communities using the Community Temperature Index, the abundance‐weighted average Species Thermal Index of all species recorded within a community, to identify areas of vulnerability where environmental temperatures diverge from the typical thermal affinities of species occurring there (e.g., Day et al., 2018; Stuart‐Smith et al., 2015).

These metrics derived from species' realized thermal niches can predict both species‐ and community‐level change in marine communities (Day et al., 2018), which is encouraging because they can be derived from data that are much more widely available than experimental data on thermal limits. Data on where marine species occur are available in large quantities from many sources (Edgar et al. 2016), with the Ocean Biogeographic Information System (OBIS, 2018) providing an open access global repository of >59M occurrence records of >120,000 marine species. Each of these occurrence records is located in space (latitude, longitude) and time (month, year), with many of them also including information on sampling depth. However, few occurrence records have in situ measures of environmental temperature associated with them, meaning that alternative sources of sea temperature need to be used to match the occurrence to environmental temperature post hoc. At present, there is no standard protocol for doing this. In addition, global occurrence datasets such as OBIS are known to contain significant and systematic spatial, temporal, and taxonomic biases (e.g., Edgar et al., 2016; Menegotto & Rangel, 2018; Miloslavich et al., 2016), which remain despite recent efforts to automate quality control (e.g., Vandepitte et al., 2015). In particular, data are biased toward well‐known species in certain groups (e.g., chordates; Miloslavich et al., 2016), with most species represented by very few occurrence records. Data are also primarily available from shallow, coastal seas in regions such as the North Atlantic (Edgar et al., 2016), with major gaps in important ecosystems like the deep pelagic ocean (Webb, Vanden Berghe, & O'Dor, 2010). Given these biases and potential sources of error at every step of the process of generating species‐level thermal affinities, it remains uncertain whether matching raw OBIS occurrence records to globally aggregated sea temperature records will actually reflect the physiological temperature limits of marine species to any degree.

Here, we use two major compilations of experimentally derived critical maximum temperatures (Bennett et al., 2018a,b; Comte & Olden, 2017) to extract upper thermal limits (based on various measures) for 533 marine species. We present a workflow to efficiently and repeatably estimate the realized thermal niche of these species by matching global occurrences extracted from OBIS to two complementary global sea temperature databases. Specifically, we use a high‐resolution global climatology of sea surface and sea bottom temperature (Assis et al., 2018; Tyberghein et al., 2012), allowing matching of occurrence records by latitude and longitude, and a depth‐resolved monthly sea temperature dataset allowing matching by latitude, longitude, sample depth, and sample date (Cheng et al., 2017; Cheng & Zhu, 2016). We calculate a range of measures of “thermal affinity,” including measures of both central tendency and upper bounds of the temperatures of a species' occurrence records. We divide species into broad functional groups, allowing us to select the most appropriate sea temperature measure (e.g., surface versus bottom temperature) to use to calculate thermal affinity. This allows us to determine the extent to which these observational, occupancy‐derived thermal affinities, which can be rapidly and automatically generated using openly available data, reflect experimentally derived thermal limits. We then use these to test whether the environmental temperature at which a species lives influences how close it is to its thermal limit, and specifically whether our data are able to detect the trend observed in more targeted studies for smaller thermal safety margins in warm‐water species (e.g., Pinsky, Eikeset, McCauley, Payne, & Sunday, 2019; Sunday et al., 2011; Waldock et al., 2019). Furthermore, by developing this workflow using open data within the open source environment R (R Core Team, 2018) in RStudio (RStudio Team, 2016), we make generating robust estimates of the thermal affinities of very large numbers of marine species generally available to the community.

## 2 METHODS

### 2.1 Experimental temperature thresholds

We obtained experimentally derived thermal thresholds from two complementary published databases. First, we used Comte and Olden (2017)'s compilation of thermal limit data for ray‐finned fish (hereafter Comte‐Olden). This dataset includes thermal limits for adult fish derived from both CT_max_ and LT_50_ studies meeting certain quality‐control criteria (e.g., including acclimation or collection temperatures; see Comte and Olden (2017) for full details). We used only the direct experimental measures of critical thermal maxima presented by Comte and Olden (2017) to avoid any circularity inherent in using their imputed data, which incorporated environmental temperatures, to test our methods. We extracted data for the 158 marine species from 17 Orders present in the Comte‐Olden dataset. All species names were matched to the World Register of Marine Species (WoRMS, WoRMSEditorialBoard, 2018) standard using the worrms package (Chamberlain, 2018) in R (R Core Team, 2018) allowing us to assign a valid WoRMS AphiaID to each name. We took the mean thermal limit for any species with >1 estimate. To give more weight to more precise estimates, we weighted this mean by the inverse of the standard deviation of each estimate (if provided). We used FishBase data (Froese & Pauly, 2018) accessed through the rfishbase package (Boettiger, Lang, & Wainwright, 2012) to classify species in the Comte‐Olden dataset as benthopelagic, demersal, bathydemersal, reef‐associated, pelagic‐neritic, or pelagic‐oceanic. Because of small numbers of species in some categories, we combined bathydemersal (*n* = 3) and demersal (*n* = 60) into a single demersal category, and pelagic‐neritic (*n* = 10) and pelagic‐oceanic (*n* = 2) into a single pelagic category. These habitat classifications were used to determine which measure of sea temperature (surface or bottom) was most appropriate for deriving the environmental thermal affinity of each species (see below).

Our second source of marine species thermal limits was the GlobTherm database (Bennett et al., 2018a,b), which includes thermal tolerance metrics for adults of over 2,000 species across all habitats and major domains of multicellular life. Most species in GlobTherm have only a single upper thermal limit value (primarily CT_max_, although a range of measures are included), which we term *T*_max_. The exceptions are some algae where both LT_0_ and LT_100_ (i.e., the temperature at which 0% and 100% of individuals are dead) are given. For these species, we selected LT_100_ as our value of *T*_max_. To identify marine species from the GlobTherm data, we matched all names to WoRMS using worrms (Chamberlain, 2018) and retained those for which we could find a WoRMS AphiaID linked to a valid marine species. We further filtered the dataset to those species known to have occurrence records in the Ocean Biogeographic Information System (OBIS, 2018), using the checklist function in the robis package (Provoost & Bosch, 2019). This resulted in a dataset of 421 marine species from 3 Kingdoms, 11 Phyla, and 24 Classes. Species from the GlobTherm dataset were assigned to functional groups using WoRMS attributes data (WoRMSEditorialBoard, 2018) accessed via the worrms package (Chamberlain, 2018) supplemented with taxonomic information and additional information from SeaLifeBase (Palomares & Pauly, 2018) accessed through the rfishbase package (Boettiger et al., 2012). The functional groups were benthos, birds, fish, macroalgae, and mammals, which allowed us to decide on appropriate sea temperature measures (surface or bottom temperature) for each group of species. In addition, we wanted to allow for different relationships between thermal limits and sea temperature‐based thermal affinities for different kinds of species (e.g., endotherms, species breeding on land). Fish were further categorized by habitat affinity as described for the Comte‐Olden data. We do not further distinguish between species in either data source, because automation of finer distinctions based, for example, on habitat is not currently possible using the sources we rely on.

### 2.2 Occupancy‐derived thermal affinities

We developed a workflow to obtain global occurrence records for each species in the experimental data list, to match these occurrences to global sea temperature datasets, and to derive summary statistics describing the realized thermal affinity (i.e., the temperatures of all the recorded global occurrences of a species). This workflow was implemented in R v3.5.1 (R Core Team, 2018) using the tidyverse v1.2.1 suite of packages (Wickham, 2017).

#### 2.2.1 Global occurrence records

We used the robis package v2.1.10 (Provoost & Bosch, 2019) to extract all global occurrence records from the Ocean Biogeographic Information System (OBIS, 2018) for all species in our dataset. First, we used the checklist() function to identify those species with records in OBIS. 157 of the 158 Comte‐Olden species and 421 GlobTherm species had at least one record in OBIS. For each of these in turn, we then used the occurrence() function to extract the latitude, longitude, depth, and date of all available records. Depth (in m) is typically recorded in OBIS as a positive value (i.e., 0 = sea surface, 100 = 100 m deep) but is not available for all records and is recorded as negative (i.e., 0 = sea surface, −100 = 100 m deep) by some sources. Because negative values can also indicate intertidal records or, in some data sources, missing values, we replaced all negative and missing depth values were with 0, effectively assuming that the species was sampled at the sea surface. Because our temperature matching process records both sea surface and sea bottom temperature, however, we are able to use the most appropriate of these measures depending on species habitats or functional groups (see below). Dates were parsed into month and year values; missing dates were not replaced. Each occurrence of an individual species was then matched to the temperature datasets described below by latitude and longitude (Bio‐ORACLE) or by latitude, longitude, depth, and date (month‐year; IAP gridded).

#### 2.2.2 Global temperature datasets

We used two complementary global sea temperature datasets. First, we used the sdmpredictors package v0.2.8 (Bosch, 2018) to access the Bio‐ORACLE database (Assis et al., 2018; Tyberghein et al., 2012) as raster layers in R. We used two Bio‐ORACLE layers, mean sea surface temperature (SST, ˚C, mean from monthly climatologies 2002–2009) and mean sea bottom temperature (SBT, ˚C, mean from monthly climatologies 2002–2014 at mean bottom depth). Both layers cover the global oceans at 5 arcmin resolution. Hereafter, we refer to these datasets as the Bio‐ORACLE temperature data. Second, we used the Institute of Atmospheric Physics' (IAP) global gridded temperature dataset (see Cheng et al., 2017; Cheng & Zhu, 2016) which provides monthly mean temperature at depth from the surface to 2,000 m since 1940 at 1 degree resolution. The depth resolution uses the standard depth bands of the World Ocean Atlas (Boyer et al., 2013) to 2,000 m, with 10 m bands to 50 m, 25 m bands from 50 to 200 m, 50 m bands from 200 to 300 m, 100 m bands from 300 to 1,500 m, and 250 m bands from 1,500 to 2,000 m. Data are provided as monthly NetCDF files which we accessed via ftp using the ncdf4 package v1.16 (Pierce, 2017). We refer to this second temperature dataset as IAP‐gridded.

#### 2.2.3 Taking the temperature of occurrence records

Occurrence records were matched to the Bio‐ORACLE and IAP‐gridded temperature datasets using a set of functions that we developed, which are fully documented in the data archive available via GitHub (https://github.com/tomjwebb/occurrence‐derived‐thermal‐affinity) and in Figshare via the University of Sheffield's Online Research Data repository (https://doi.org/10.15131/shef.data.12249686). These in turn use the packages sp v1.2.5, rgdal v1.2.11, and raster v2.9.5 (Bivand, Keitt, & Rowlingson, 2018; Bivand & Pebesma, 2005; Hijmans, 2017; Pebesma & Bivand, 2005). For Bio‐ORACLE, temperature values were extracted from the SST and SBT rasters for each occurrence referenced by latitude and longitude. We also assigned a “best” temperature for each record, based on the species' habitat or functional group, taking SST for birds, mammals, and pelagic and reef‐associated fish, and SBT for benthos, macroalgae, and demersal and benthopelagic fish. For IAP‐gridded, occurrence records from within the time period covered by the dataset (1940–2017) were matched by latitude, longitude, date (month‐year), and depth. Three temperature values were extracted for each occurrence: SST, SBT (actually the temperature at the deepest available depth layer, equivalent to SBT in seas <2,000 m, temperature at 2,000 m otherwise; hereafter referred to simply as SBT), and temperature at sampling depth (i.e., the temperature at the depth recorded for the species occurrence). Given the relatively coarse spatial resolution of the IAP dataset, some occurrences were unable to be matched at all (e.g., if most of the 1‐degree cell fell on land), and some sample depths were deeper than the mean depth over the 1‐degree cell, meaning that T at depth was sometimes unavailable even though SST and SBT were. Occurrences for which we could not obtain a temperature match were excluded from calculations of thermal affinity.

#### 2.2.4 Species‐level occupancy‐derived thermal affinity

The thermal affinity of a species was calculated on the basis of the temperature‐matched occurrence records. For each species, we calculated the mean, minimum, maximum, median, standard deviation, median absolute deviation, and 5th and 95th quantiles of each temperature measure, to capture different regions of the underlying thermal performance curve. These summary metrics were typically highly positively correlated with each other (see below), and so to simplify presentation of results, we focus on mean temperature of occurrences as our primary measure of a species' thermal affinity. We also recorded for each species the total number of occurrences and the number that we were able to match to each temperature dataset.

### 2.3 Statistical analysis

For each species, we calculated two thermal affinity measures (mean and the 95th quantile of observed matched temperatures) derived from Bio‐ORACLE (SST, SBT, and “best” Bio‐ORACLE temperature) and from IAP temperature at depth. We calculated correlations between each of these measures across all species. We explored flexible smoothing methods (GAMs) for the relationships between experimentally derived thermal maximum and occurrence‐derived mean thermal affinity, but in all cases, the smoothed fits did not indicate relationships markedly more complex than simple linear or unimodal curves. For ease of interpretation, especially when including interactions between continuous and categorical predictions, we therefore modeled experimentally derived thermal maximum as a second order polynomial function of occurrence‐derived mean thermal affinity, calculated using both “best” Bio‐ORACLE temperature and IAP temperature at depth. For the Comte‐Olden dataset, we included fish habitat group as a fixed effect, and for the GlobTherm dataset, we included functional group, also as a fixed effect. We tested for an interaction of this grouping variable with thermal affinity and compared the overall fit of interactive and additive models as well as models excluding this variable. Finally, we pooled data across Comte‐Olden and GlobTherm datasets and for each species calculated the difference between experimentally derived thermal maximum and mean thermal affinity derived from occurrence data (using IAP temperature at depth). We modeled this relationship as a second order polynomial function (experimentally derived thermal maximum ~ poly(occupancy‐derived thermal affinity, 2)), separately for the major groups (benthos, macroalgae, pelagic, reef‐associated and demersal fish) to test whether species occurring at low, high, or intermediate temperatures vary in how close they live to their thermal limits.

Processed datasets and code for analysis and visualization are available via GitHub (https://github.com/tomjwebb/occurrence‐derived‐thermal‐affinity) and are also deposited in Figshare via the University of Sheffield's Online Research Data repository, https://doi.org/10.15131/shef.data.12249686.

## 3 RESULTS

**FIGURE 1:**
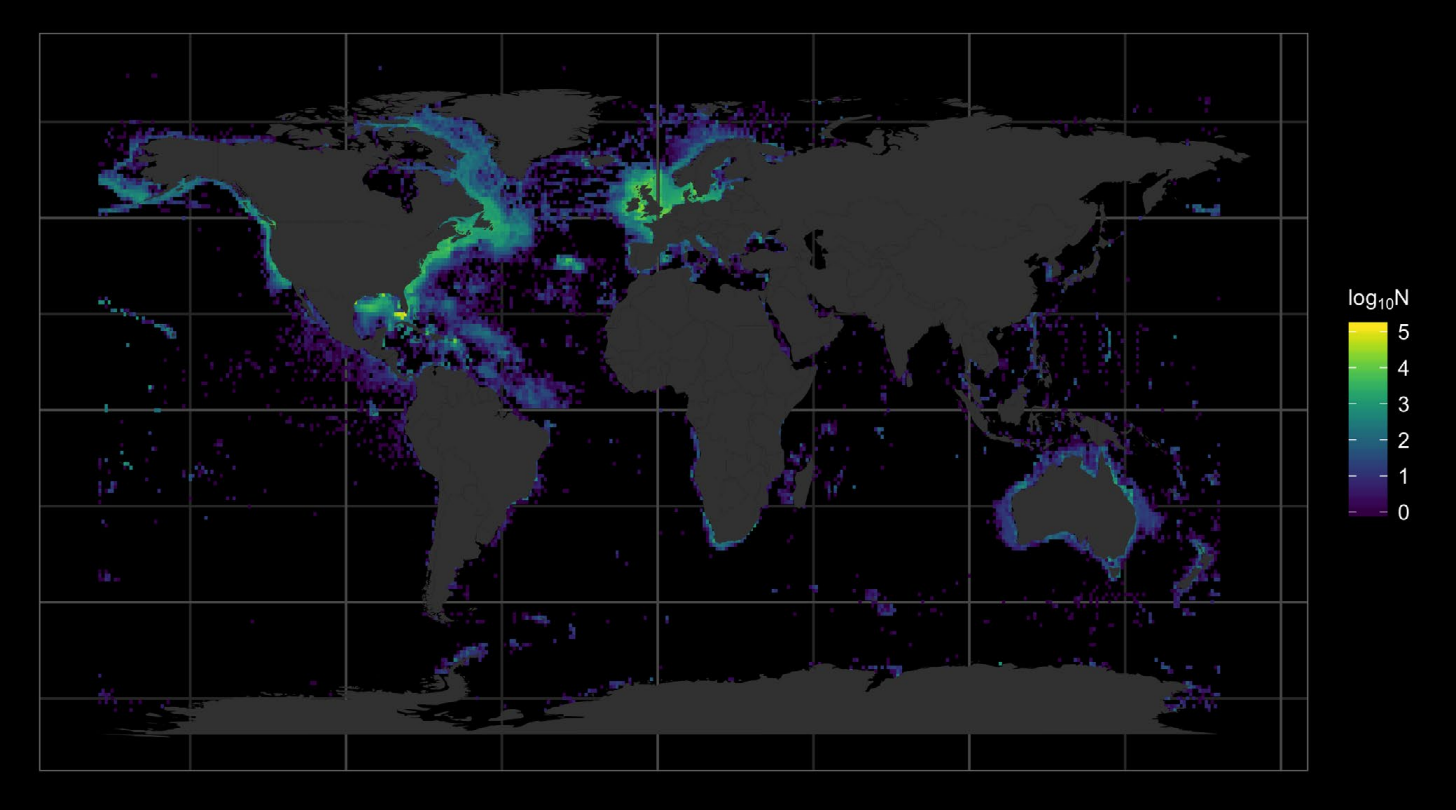
The global distribution of the 2,176,906 occurrence records obtained from OBIS for 533 marine species with experimentally derived thermal maxima available from our two data sources (Bennett et al., 2018a,b; Comte & Olden, 2017), mapped on a 1° grid

All but one of the 158 fish species in the Comte‐Olden experimental dataset (63 demersal, 17 benthopelagic, 12 pelagic, 66 reef‐associated) and all 421 species from GlobTherm dataset (85 benthos, 9 birds, 82 fish, 235 macroalgae, 5 mammals, 5 nekton) are present in OBIS with a combined total of 495,795 and 1,821,183 occurrence records, respectively, meeting our quality requirements. Forty‐five species are present in both datasets, taking these into account results in a total of 2,176,906 occurrence records across 533 valid marine species. These records span the years 1643–2017, 0‐5,870 m depth, 81.1°S to 88.8°N, and 180°W to 180°E—although most records are from coastal North America, NW Europe, Southern Africa, and Australasia (Figure 1). The experimental maximum temperatures reported for the 44 species shared between the two datasets are highly positively correlated (*r* = .96) but not identical, and so we include these species in our analyses of both datasets.

Across both sets of species, most (>96%) of occurrences records were successfully matched to a Bio‐ORACLE temperature (Table 1). Somewhat fewer were successfully matched to the IAP‐gridded data (68.4%–83.7%; Table 1), primarily due to missing date information (95,320 records) or years out of the IAP range (17,596 records). However, a temperature affinity based on all temperature measures was available for almost all (>95%) species, regardless of data source (Table 1).

**TABLE 1:** Number and percentage of the 2,176,906 total OBIS records and 533 species which were successfully matched to each temperature measure using Bio‐ORACLE and IAP‐gridded temperature data, shown separately for the two thermal maxima databases

Temperature affinity measure	Comte olden data (*N* = 157 species)		Globtherm data (*N* = 421 species)	
	Number (%) matched records	Number (%) matched species	Number (%) matched records	Number (%) matched species
Bio‐ORACLE SST	476,213 (96.1%)	157 (100%)	1,753,998 (96.3%)	420 (99.8%)
Bio‐ORACLE SBT	476,217 (96.1%)	157 (100%)	1,733,619 (96.3%)	420 (99.8%)
IAP‐gridded SST	365,627 (73.8%)	154 (98.1%)	1,523,531 (83.7%)	403 (95.7%)
IAP‐gridded SBT	365,627 (73.8%)	154 (98.1%)	1,523,531 (83.7%)	403 (95.7%)
IAP‐gridded T	338,883 (68.4%)	154 (98.1%)	1,362,461 (74.8%)	403 (95.7%)

Temperature affinity calculated using different summary measures and different temperature data sources was typically highly positively correlated. Across all temperature data sources, the correlations between the mean and 95% quantile temperature affinities of species were almost always >0.90. Affinities based on SST from Bio‐ORACLE versus IAP were very similar (*r* > .9). Using SBT resulted in somewhat weaker correlations between data sources and between summary measures within data sources (0.75–0.80). Affinities calculated using temperature at depth from IAP and “best” Bio‐ORACLE temperature (using SBT for benthopelagic, demersal, and bathydemersal fish and for benthos and macroalgae, and using SST for reef‐associated, pelagic‐neritic, and pelagic‐oceanic fish and for birds and mammals) were very tightly correlated (*r* > .95 in all cases). We focus here on mean temperature affinities derived from the “best” Bio‐ORACLE source (SST or SBT depending on group as described above) and temperature at sample depth from the IAP gridded dataset, but given the strong correlations between temperature metrics our conclusions are not substantially affected by this choice. Hereafter, we refer to the “best” Bio‐ORACLE sources simply as Bio‐ORACLE, and IAP temperature at sample depth as IAP gridded.

**FIGURE 2:**
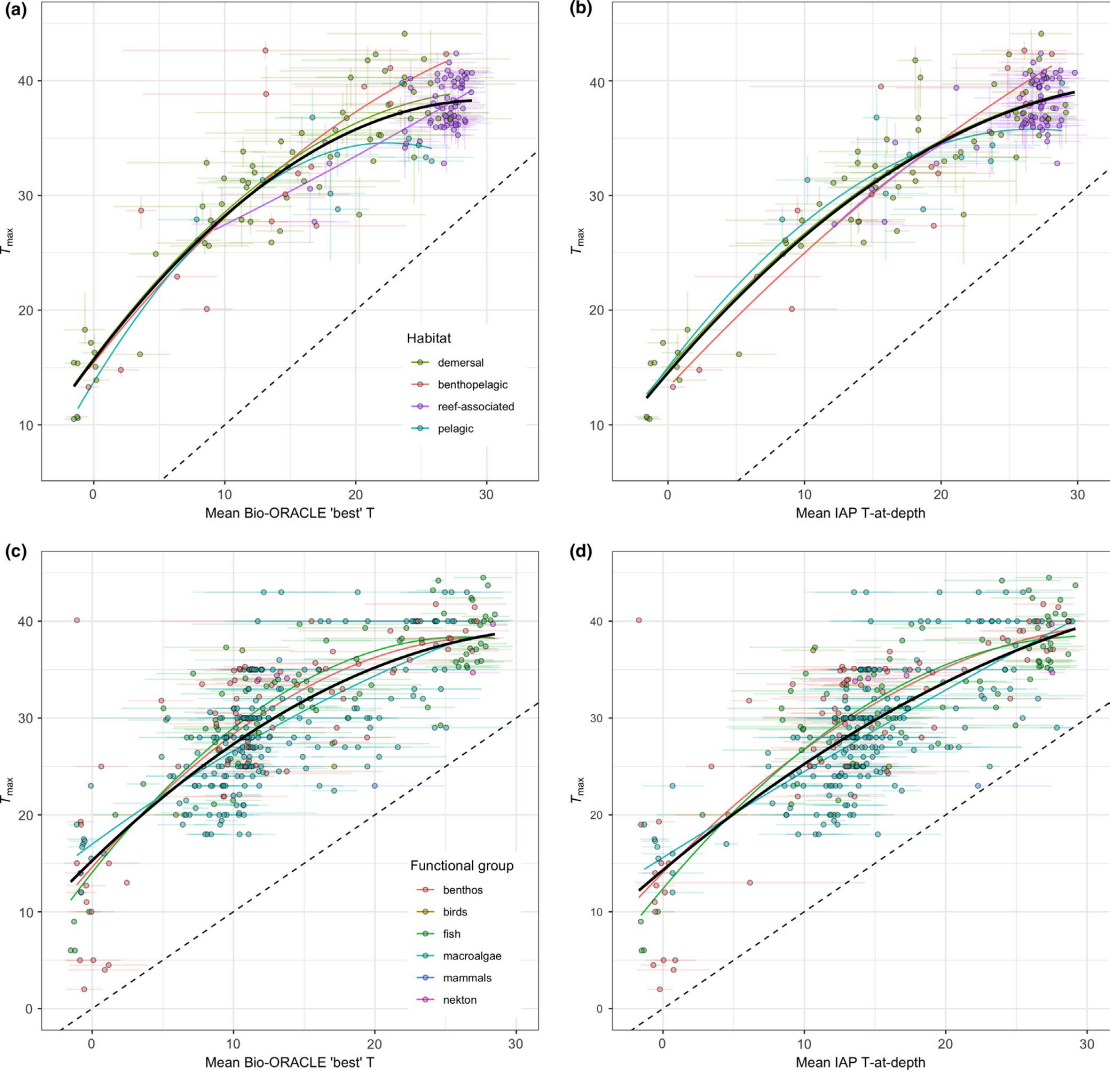
Experimentally derived critical thermal limits (*T*_max_) for 157 (a) and 154 (b) marine fish species taken from (Comte & Olden, 2017), and for 420 (c) and 403 (d) marine species taken from the GlobTherm (Bennett et al., 2018a,b), against occupancy‐derived thermal affinity calculated from a combination of Bio‐ORACLE sea surface temperature (SST) and sea bottom temperature (SBT) depending on fish habitat or functional group, at c. 9 km resolution (a, c), and date (month‐year)‐matched IAP gridded temperature at sample depth at 1° resolution (b, d). Points are means, and error bars are min and max reported values for *T*_max_ (where available) and standard deviations for temperature affinities. In each case, separate 2nd order polynomials are shown for each of the habitat associations (a, b) or functional groups (c, d) (colored lines), together with a single 2nd order polynomial fitted across all species (solid black line). The 1:1 relationship is shown as a dashed line

Species‐level thermal affinities derived from both Bio‐ORACLE and the IAP gridded dataset are both strongly positively related to experimentally derived *T*_max_ with relationships generally unimodal with a peak at intermediate to high temperature affinities (Figure 2). These relationships were also highly consistent across habitats and functional groups. For the Comte‐Olden dataset, there was no evidence of an interaction between Bio‐ORACLE temperature and habitat (*df* = 6, 145, *F* = 1.23, *p* = .2938, Figure 2a), and while refitting without the interaction reveals habitat to be significant (*df* = 3, 151, *F* = 3.057, *p* = .0302), model multiple R^2^ increases only marginally (from 0.83 excluding habitat to 0.84 including it). There is some weak evidence for higher *T*_max_ at a given thermal affinity in demersal (intercept: 15.9°C, 95% CI: 14.2–17.5) and benthopelagic species (16.2°C, 14.2–18.2) than in pelagic species (13.4°C, 11.1–15.7), with reef‐associated species intermediate (14.4°C, 12.4–16.5); however, these differences are small. Using IAP temperature at depth to calculate thermal affinity (Figure 2b), again there is no evidence of an interaction between habitat and thermal affinity (*df* = 6, 142, *F* = 1.17, *p* = .328); in this case, there is no main effect of habitat either (*df* = 3, 148, *F* = 0.25, *p* = .861), and including habitat has minimal effect on the overall model *R* ^2^ (*R* ^2^ excluding habitat = 0.86, including habitat = 0.87). For both thermal affinity measures, *T*_max_ increases over the full range of estimated thermal affinities with the polynomial suggesting a maximum value of *T*_max_ at a Bio‐ORACLE thermal affinity of 33.4°C and an IAP thermal affinity of 36.9°C.

Initial examination of the relationship between *T*_max_ and thermal affinity for the species in the GlobTherm dataset revealed two extreme outliers, the oribatid mite *Halozetes belgicae* (Michael, 1903) (WoRMS Aphia ID 508323), which has *T*_max_ recorded in GlobTherm as 40.1°C but for which we estimate mean thermal affinities as −1.08°C (Bio‐ORACLE) to −1.71°C (IAP), albeit on the basis of just 2 OBIS records; and the red alga *Devaleraea ramentacea* (Linnaeus) Guiry, 1982 (WoRMS Aphia ID 145770), which has *T*_max_ recorded in GlobTherm as 50°C but for which we estimate mean thermal affinities as 6.4°C (Bio‐ORACLE) to 11.4°C (IAP). Checking the original references cited in GlobTherm, we find that the critical maximum temperature of *H. belgicae* is as reported in Deere, Sinclair, Marshall, and Chown (2006), but these authors report this species as occurring in the supra‐littoral zone, so although it is reported as “marine” by WoRMS, air temperature is probably more appropriate than sea temperature for this species. This may be the case for other species in our dataset, however, so we retain *H. belgicae* in our analyses, but note that it has minimal impact on our statistical results or conclusions. The original cited reference for *D. ramentacea* in GlobTherm (Bischoff & Wiencke, 1993) records the maximum survival temperature of this species as 19°C, and so we change its *T*_max_ to 19°C for our analyses, although we note this decision too has no influence on our overall conclusions. We also exclude birds, mammals, and nekton from our discussion of GlobTherm species as there are few species (9, 5, and 5, respectively).

Using Bio‐ORACLE thermal affinity, we find evidence of a significant interaction between functional group and the second order polynomial term for thermal affinity (*df* = 8, 384, *F* = 2.20, *p* = .0271): the quadratic coefficients for benthos (−0.029, 95% CI: −0.042 to −0.015) and fish (−0.035, −0.054 to −0.015) are both significantly negative whereas that for macroalgae (−0.010, −0.028 to 0.009) does not differ significantly from 0. However, this model (*R* ^2^ = .65) explains only marginally more variance than models excluding the interaction (*R* ^2^ = .63) or excluding functional group altogether (*R* ^2^ = .62), indicating that the statistically significant differences between functional groups in the relationship between thermal affinity and *T*_max_ represent only a small amount of variation around the overall pattern across functional groups (Figure 2c). Similar results are found when using IAP thermal affinity, where there is a significant interaction between functional group and the second order polynomial term for thermal affinity (*df* = 8, 367, *F* = 2.26, *p* = .0231): the quadratic coefficients for benthos (−0.021, −0.034 to −0.019) and fish (−0.029, −0.047 to −0.010) are both significantly negative whereas that for macroalgae (−0.002, −0.019 to 0.014) does not differ significantly from 0. Again, including these interactions (*R* ^2^ = .66) explains only marginally more variance than models excluding the interaction (R^2^ = 0.64) or excluding functional group altogether (*R* ^2^ = 0.62) (Figure 2d).

**FIGURE 3:**
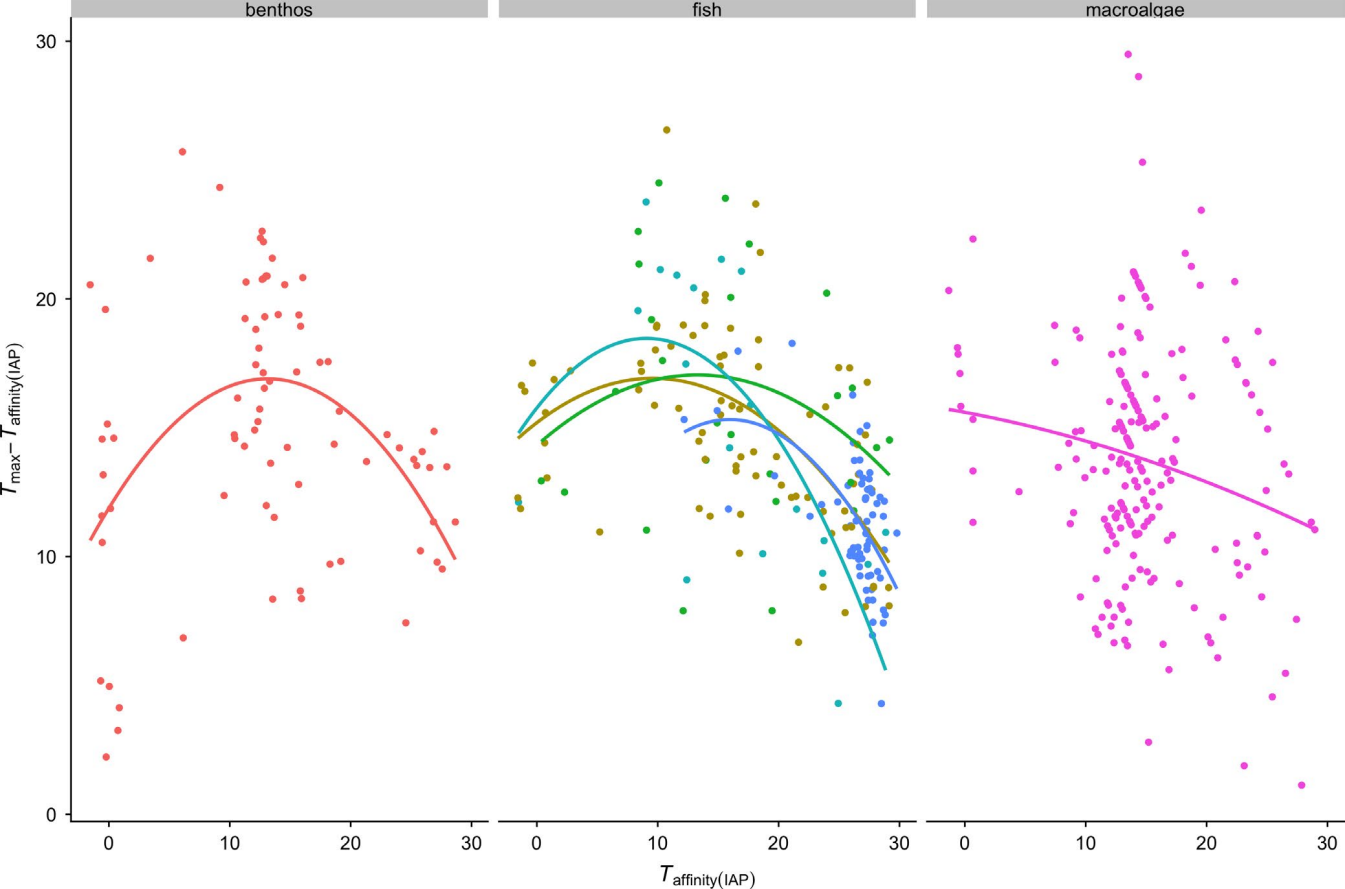
Difference between occupancy‐derived temperature affinity and *T*_max_ across benthos, fish, and macroalgae, as a function of IAP T at depth temperature affinity. These figures combine *T*_max_ data from both the Comte‐Olden and GlobTherm databases. Fish are further divided into demersal (khaki), benthopelagic (green), pelagic (light blue), and reef‐associated (dark blue) species. Lines are fits from second order polynomial models

The unimodal relationships illustrated in Figure 2 imply that species with intermediate thermal affinities tend to live further from their *T*_max_. This is shown more clearly in Figure 3, where, combining data from both Comte‐Olden and GlobTherm databases, the difference between experimentally derived *T*_max_ and occupancy‐derived thermal affinity clearly declines with increasing *T*_affinity_ in benthos, fish, and macroalgae, from around 14–17°C in species with a mean *T*_affinity_ of between 10 and 20°C, to around 9–12°C in species with a mean *T*_affinity_ > 20°C (Table 2). In benthos in particular, there is also a clear decline at low *T*_affinity_ (difference of around 12°C in species with a mean *T*_affinity_ of < 10°C; Table 2) indicating that species living in extremely cold and extremely warm water tend to be closer to their thermal maxima. Further statistical details are not given for the curves shown in Figure 3 as for benthos and macroalgae, they are simply the same curves as in Figure 2d offset by IAP T at depth, and for fish, they are very similar to the curves in Figure 2b offset by IAP T at depth.

**TABLE 2:** Difference (mean ± *SD*) between *T*_max_ and mean occupancy‐derived temperature affinity (IAP T at depth) across functional groups, for species with a cold (≤10°C), moderate (>10°C and ≤20°C), and warm (>20°C) temperature affinity

Functional group	Difference between *T*_max_ and mean temperature affinity		
	Mean *T*_affinity_ ≤ 10°C	10°C < Mean *T*_affinity_ ≤ 20°C	Mean *T*_affinity_ > 20°C
Benthos	12.7 ± 7.21°C	16.6 ± 3.99°C	12.4 ± 2.26°C
Reef fish	NA	16.5 ± 3.61°C	12.0 ± 3.05°C
Demersal fish	15.9 ± 2.40°C	16.1 ± 5.66°C	15.2 ± 2.79°C
Benthopelagic fish	16.6 ± 4.60°C	17.2 ± 4.70°C	9.46 ± 2.68°C
Pelagic fish	18.5 ± 5.90°C	14.8 ± 2.38°C	11.1 ± 2.34°C
Macroalgae	15.2 ± 3.39°C	14.0 ± 4.36°C	11.5 ± 5.00°C

## 4 DISCUSSION

Our analyses show that across 533 marine species, measures of thermal affinity derived from matching global occurrence records obtained from the Ocean Biogeographic Information System (OBIS, 2018) to global sea temperature datasets (Assis et al., 2018; Cheng et al., 2017; Cheng & Zhu, 2016; Tyberghein et al., 2012) are highly significantly associated with independent, experimentally derived thermal maxima (*T*_max_) (Figure 2). The species in our dataset represent 24 taxonomic classes occupying a wide range of benthic, pelagic, and coastal habitats throughout the global oceans (Figure 1), with *T*_max_ spanning 2–44.5°C, suggesting a truly general relationship. This is an important and encouraging result: The expense of experimental studies of thermal tolerance (e.g., Pacifici et al., 2015) means that the total number of marine species experimentally assessed is unlikely to substantially exceed the 533 already compiled by (Comte & Olden, 2017) and in the GlobTherm database (Bennett et al., 2018a,b), whereas many more species have occurrences recorded in OBIS (currently >120,000 species; OBIS, 2018). This further validates previous studies which have used occurrence‐based temperature affinities of marine species to generate indices such as the Community Thermal Index to monitor climate‐driven changes to marine communities (Bates et al., 2014; Stuart‐Smith et al., 2015), and our simple workflow provides opportunities to address issues of limited taxonomic representation in studies of climate vulnerability (e.g., Pacifici et al., 2015) by deriving estimates of thermal affinity over whole assemblages even in the absence of systematic surveys (see Webb and Lines (2018) for an example application to >26,000 European marine species, using a parallelized version of the workflow documented here).

The strength of the relationship between thermal affinity and *T*_max_ is perhaps surprising given that our method of matching occurrence records to global gridded temperature products introduces a number of potentially major sources of error. First, global temperature products derived from downscaling or interpolating from instrumental measurements may not accurately reflect the ambient temperatures actually experienced by organisms. On land, these errors can be on the order of several °C (Roberts, Wood, & Marshall, 2019), and although discrepancies are likely to be reduced in the sea due to greater spatial and temporal autocorrelation in temperature (e.g., Steele, 1985; Sunday et al., 2011; Webb, 2012), and consequently less pronounced microclimates or potential for behavioral temperature regulation (Sunday et al., 2011, 2014), it is still likely that the grid‐scale temperature value assigned to each occurrence record is an imprecise estimate of the temperature experienced by that organism—in particular for organisms occupying certain habitats, such as the intertidal zone, that our workflow does not currently discriminate. Such effects will differ between the two temperature datasets we used, which had contrasting strengths and weaknesses. They differ in their spatial resolution, and in whether they include depth and time, with the Bio‐ORACLE data (Assis et al., 2018; Tyberghein et al., 2012) constituting a time‐averaged climatology with values available only for sea surface and sea bottom temperature, although at a higher spatial resolution (5 arcmin) than the gridded IAP data (Cheng et al., 2017; Cheng & Zhu, 2016), which includes temperature at depth as well as seasonal (monthly) and annual variation on a 1 degree grid.

Despite these contrasts in the structure of the temperature data, there were no major differences in the thermal affinities produced: Mean temperature affinities derived from “best” Bio‐ORACLE temperature (SST or SBT depending on species habits) and IAP temperature‐at‐sample‐depth are both strongly correlated with species *T*_max_, with the IAP temperature affinity providing little more explanatory power (C‐O: *R* ^2^ = .86–.87; GlobTherm: 0.62–0.66) than the “best” Bio‐ORACLE temperature affinity measure (C‐O: *R* ^2^ = .83–.85; GlobTherm: 0.62–0.65). Our results do not therefore appear to be particularly sensitive to small errors in environmental temperature estimates. This suggests that obtaining and matching occurrences to time‐ and depth‐resolved temperature data may not be worth the additional computational time incurred. However, it is worth noting that sample depth information was missing for almost half of all the occurrence records (>1 M), for all of which we assumed depth = 0 m. Therefore, the lack of extra information provided by the IAP temperature‐at‐sample depth could be a result of losing the extra dimension of variability in ocean temperature provided by depth. Equally, although occurrences in our dataset do span a wide range of depths, most were from shelf seas (Figure 1), and it is possible that temperature at depth would prove more useful in open ocean and deep‐sea ecosystems.

Globally, trading off spatial and temporal resolution, as seen in Bio‐ORACLE versus IAP gridded, seems inevitable. However, for some regions, datasets with both depth and high spatial resolution are available. For example, 1981–2015 hindcasts from the coupled NEMO‐ERSEM physical oceanographic and biogeochemical models (Butenschon et al., 2016) provide depth‐resolved temperature at high spatial (<10 km) and temporal (<monthly) resolutions for the North Western European Shelf and part of the North East Atlantic, enabling more precise matching of species occurrences to environmental temperature. However, species occurrences from outside the modeled region cannot be included in estimates of species temperature affinity, potentially limiting the generality of such methods. A detailed analysis of the costs and benefits of using different temperature datasets would be a valuable exercise; however, our results suggest that a computationally efficient way to obtain a robust estimate of temperature affinity for large numbers of species is to use a simple climatology such as Bio‐ORACLE (Assis et al., 2018; Tyberghein et al., 2012).

The strength of the relationship between temperature affinity and *T*_max_ is also surprising given the known biases in taxonomic, spatial and temporal coverage of OBIS (e.g., Edgar et al., 2016; Miloslavich et al., 2016; Webb et al., 2010), such that the occurrence records we use to calculate a species' temperature affinity will typically only constitute a small and nonrandom sample of the locations at which the species actually occurs. Although our dataset comprised almost 2.2 million occurrence records in total, most species are represented by relatively few occurrence records (median number of OBIS records = 360 for Comte‐Olden species, 177 for GlobTherm species), making the ability to relate temperature affinity based on such a small number of occurrences to *T*_max_ even more encouraging. It is worth noting, however, that the species for which we have *T*_max_ estimates are very likely better‐studied, and more coastal or shallow‐dwelling (Figure 1), than most marine species. The similarity of relationships across diverse functional and taxonomic groups, though, gives us some confidence in applying our method to a much wider range of species, and the benefits of using a global, open access database such as OBIS should not be overlooked.

Finally, the database of experimentally derived thermal limits that we use includes data derived using a range of metrics and methodologies, from species with different historical exposures to different temperature regimes and from experiments conducted often decades ago (i.e., when species were living in a different global thermal environment), all of which can affect how thermal limits are perceived to covary with environmental factors (Sunday et al., 2019). These methodological differences no doubt account for some of the residual variation observed in our analyses, but different measures of *T*_max_ are likely to be highly correlated within species and our results suggest that environmentally derived thermal affinity correlates well with all of them.

Another important pattern to emerge from our analyses is that across functional groups, species living in warmer waters (higher *T*_affinity_) are closer to their experimentally derived thermal maximum than those with intermediate thermal affinities (Figure 3). For fish and benthos, this is also true of species living in colder waters (Figure 3). Previous work has shown that the thermal tolerance breadth of marine species typically highest at midlatitudes (Sunday et al., 2011), and it appears to be a general pattern that tropical species live nearer to their upper thermal limits (i.e., have a smaller “thermal safety margin”; Pinsky et al., 2019; Waldock et al., 2019), perhaps because they are adapted to a less variable thermal regime than species in temperate systems (Sunday et al., 2019). The extent of the nonlinearity evident in our data is rather small, but does add to the evidence that warming is likely to have particularly severe effects for thermal specialists in the tropics (Bruno et al., 2018; Lough, Anderson, & Hughes, 2018; Pinsky et al., 2019; Rummer & Munday, 2017; Tewksbury, Huey, & Deutsch, 2008).

In conclusion, our simple method of matching openly available marine species occurrence records to openly available global sea temperature products results in estimates of occupancy‐derived species‐level thermal affinity that correlate strongly with experimentally derived critical thermal maxima and that reproduce patterns (e.g., smaller thermal safety margins in warmer water species) previously documented in more targeted studies. The workflow we propose is replicable and easily extended to further discriminate between species groups, or to encompass alternative temperature products or additional environmental variables to derive estimates of species' affinities to salinity, dissolved oxygen, benthic habitats, etc. Our work highlights the value of openly available global datasets of species occurrences and environmental variables and builds on code developed within the open source community. Our method involves approximations using imperfect data at every stage, however, given the pace and magnitude of current climate change, making best use of available data and incorporating as many species as possible into predictions of ecological responses to warming seas is imperative.

## CONFLICTS OF INTEREST

The authors declare no conflicts of interest.

## AUTHOR CONTRIBUTION

**Thomas J. Webb:** Conceptualization (lead); Data curation (lead); Formal analysis (lead); Funding acquisition (lead); Investigation (lead); Methodology (equal); Project administration (lead); Resources (lead); Software (equal); Supervision (lead); Validation (equal); Visualization (equal); Writing‐original draft (equal); Writing‐review & editing (lead). **Aaron Lines:** Conceptualization (supporting); Data curation (supporting); Formal analysis (supporting); Investigation (supporting); Methodology (supporting); Software (equal); Writing‐original draft (equal); Writing‐review & editing (supporting). **Leigh M. Howarth:** Conceptualization (supporting); Investigation (supporting); Methodology (supporting); Software (supporting); Writing‐original draft (supporting); Writing‐review & editing (supporting).

## Data Availability

A major aim of this work is to make the tools required to replicate, adapt, and extend the methods presented freely available to the community. Our work uses existing publicly available data, and we show users how to access the same data from within the open source statistical environment R. Processed datasets and code for analysis and visualization are available via GitHub (https://github.com/tomjwebb/occurrence‐derived‐thermal‐affinity) and are also deposited in Figshare via the University of Sheffield's Online Research Data repository, https://doi.org/10.15131/shef.data.12249686.
