# Supplementary material for: Occupancy‐derived thermal affinities reflect known physiological thermal limits of marine species
Source: Ecol Evol. 2020 Jun 15;10(14):7050–61. doi: 10.1002/ece3.6407 (PMC7391554; doi:10.1002/ece3.6407)
Supplement: Supplementary file 1 [file ECE3-10-7050-s001.pdf]

Thermal affinities and thermal limits of marine species: Supplementary Materials

Tom Webb

Aaron Lines

Leigh Howarth

01/07/2019

Supplementary Materials for Webb et al., *Environmentally-derived thermal affinities accurately predict the physiological thermal limits of marine species*, submitted for review to Ecology Letters.

The code below was developed using R version 3.5.2 (2018-12-20), Platform: x86_64-apple-darwin15.6.0 (64-bit), Running under: macOS Mojave 10.14.4. In addition, the following packages need to be loaded:

```
library(tidyverse) #v1.2.1
library(worrms) #v0.3.2
library(rfishbase) #v3.0.4
library(robis) #v2.1.10
library(lubridate) #v1.7.4
library(raster) #v2.9-5
library(sdmpredictors) #v0.2.8
library(naniar) #v0.4.2
library(ncdf4) #v1.16
library(ggcorrplot) #v0.1.1
library(cowplot) #v0.9.4
library(viridis) #v0.5.1
library(maps) #v3.3.0
```

Shortcuts: Direct loading of temperature-matched data

The following few hundred lines document how we processed the thermal limits datasets that we obtained for fish from Comte & Olden 2017, https://doi.org/10.1038/nclimate3382 (https://doi.org/10.1038/nclimate3382), and for a wide range of species from the GlobTherm Database, described in Bennett et al. (2018a, https://doi.org/10.1038/sdata.2018.22 (https://doi.org/10.1038/sdata.2018.22)) with the data itself published in Dryad (Bennett et al. 2018b, https://doi.org/10.5061/dryad.1cv08 (https://doi.org/10.5061/dryad.1cv08)) - inlcuding taxonomy checking, adding habitat affinities or broad functional groups, obtaining global occurrence records from OBIS and matching these to global sea temperature datasets prior to summarising the thermal affinities of each species. To circumvent this lengthy process, we provide the following fully-processed datasets that can be loaded directly and used to reproduce our results and figures: `t_matched_co_exp_dat_full.csv` is the

Comte-Olden data with temperature affinities added for each species, and `t_matched_globtherm_dat_full.csv` is the Globtherm data with added temperature affinities. To read these in directly so that you can proceed straight to the Analysis and Visualisation section:

```
co_exp_dat <- read_csv("data/t_matched_co_exp_dat_full.csv")
globtherm_dat <- read_csv("data/t_matched_globtherm_dat_full.csv")
```

Temperature Matching Functions

This set of 6 core functions performs the temperature-matching of occurrence records. The input is the Aphia ID (standard valid taxonomic ID from WoRMS, the World Register of Marine Species, http://www.marinespecies.org (http://www.marinespecies.org)) for a species of interest (see documentation for `worrms::wm_name2id` for a way to get the Aphia ID of a species from its scientific name). Given only the species ID, these functions get all of its global occurrence records from OBIS, the Ocean Biogeographic Information System https://obis.org (https://obis.org), download relevant global gridded sea temperature measures, and summarise the temperature across the species' occurrence records using a range of summary statistics. The code has sensible defaults for organising and looking for files, which you can use by setting:

```
use_defaults <- TRUE
```

This is recommended. The alternative is to set a bespoke filepath to used for saving datafiles obtained and generated in the code to disk as well as for checking for pre-existing files (i.e. if you have previously run the code). Set is by adding your preferred path using something like `bespoke_path <- "/Volumes/Shared/shefmeme2/Shared"` - NB - this may not be entirely consistently implemented in the code below - I recommend leaving `use_defaults` as `TRUE`.

Overall temperature-matching function

This first function is a wrapper function for getting global OBIS records for a single species, matching these to the temperature datasets, and summarising the results. The arguments are `sp_id`, which should be a valid WoRMS Aphia ID for a species known to occur in OBIS (examples of how to obtain and check this are given below). The default `save_all_recs` means that the full set of occurrence records, matched to the various temperature datasets, for this species will be saved to a CSV file. This is useful if you think you may want to use the data for another purpose, or summarise in a different way. It is also useful if you think you might be running this code multiple times - setting `check_match` to `TRUE` means that the function will check for the existence of a temperature-matched CSV file before proceeding; this can save a lot of time. `use_defaults` is as described above, and I recommend leaving this as `TRUE`. The function returns a summary of temperature affinity of the species as a `tibble`.

```
get_temp_summ_by_sp <- function(sp_id, save_all_recs = TRUE, check_match = TRUE, u
se_defaults = TRUE){
# function to get OBIS records for a given species and match to IAP and Bio-Oracle
temperatures
# if save_all_recs == TRUE, this full matched dataset will be saved before summari
sing
# function returns a summary of temperature affinity of the species
```

```
    # First, check if this species has previously been matched, if check_match is
TRUE
    # (This is an option in case the user wants to update all records)

  if(check_match == TRUE){
      out_path <- ifelse(use_defaults, paste0(file.path(getwd()), "/t_matched_obis
_recs"),
                         paste0(bespoke_path, "/t_matched_obis_recs"))
      # set species_done flag to FALSE
      species_done <- FALSE

      # check if the directory exists:
      if(dir.exists(file.path(out_path))){
        # get list of aphia ids from the files in this directory:
        done_files <- list.files(path = out_path)
        done_aphias <- done_files %>%
          word(sep = "_") %>%
          str_replace(pattern = "aphia", replacement = "") %>%
          as.integer()

        # check if species sp_id is in this list
        species_done <- sp_id %in% done_aphias
        }

      # get the file if it exists
      if(species_done == TRUE){
        which_file <- which(done_aphias == sp_id)

        # what if more than one file matches?
        if(length(which_file) > 1){
          # find the most recent one
          which_file <- which_file[order(done_files[which_file], decreasing = TRUE
) == 1]
          }

        sp_temp <- read_csv(file = paste(out_path, done_files[which_file], sep = "
/"),
                            col_types = cols(
                              decimalLongitude = col_double(),
                              decimalLatitude = col_double(),
                              depth = col_double(),
                              eventDate = col_datetime(format = ""),
                              scientificName = col_character(),
                              aphiaID = col_integer(),
                              year = col_integer(),
                              month = col_character(),
                              depth0 = col_double(),
                              grid_depth = col_integer(),
                              grid_lon = col_integer(),
                              grid_lat = col_integer(),
                              iap_t = col_double(),
                              iap_sst = col_double(),
                              iap_sbt = col_double(),
                              iap_grid_bottom_depth = col_integer(),
```

```
                                 bo_sst = col_double(),
                                 bo_sbt = col_double()
                                 )) %>%
          t_summary() %>%
          mutate(species_id = sp_id) %>%
          dplyr::select(species_id, everything())
        } else {
          sp_temp <- get_obis_recs(species_id = sp_id) %>%
            get_iap_gridded_t() %>%
            get_bio_oracle_t() %>%
            save_full_recs(save_recs = save_all_recs) %>%
            t_summary() %>%
            mutate(species_id = sp_id) %>%
            dplyr::select(species_id, everything())
          }
      } else {
        sp_temp <- get_obis_recs(species_id = sp_id) %>%
          get_iap_gridded_t() %>%
          get_bio_oracle_t() %>%
          save_full_recs(save_recs = save_all_recs) %>%
          t_summary() %>%
          mutate(species_id = sp_id) %>%
          dplyr::select(species_id, everything())
        }

  sp_temp
  }
```

Getting OBIS records

This function gets OBIS records for a given valid WoRMS Aphia ID (argument `species_id`). You can also ask this function to check that the Aphia ID actually occurs in OBIS using `missing_check`, but it is recommended to do this prior to calling these functions (see examples below). It returns a tibble of all OBIS records for the species.

```
get_obis_recs <- function(species_id, missing_check = FALSE,
                          fields = c("decimalLongitude", "decimalLatitude",
                                     "minimumDepthInMeters", "maximumDepthInMeters
", "depth",
                                     "eventDate", "year", "month",
                                     "scientificName", "aphiaID")
){
  # Fuction to get OBIS records for a given species_id, which must be a recognised
WoRMS Aphia ID

  # NB OBIS returns records from all taxa gathered under the same valid Aphia ID;
the aphia ID returned is that of the taxon as recorded, not necessarily the valid
ID, so in order that the final dataset is correctly named we add back in the 'corr
ect' ID here as valid_AphiaID

  if(missing_check == TRUE){
    # catch invalid / unrecognised AphiaIDs here - but recommend doing this prior
```

```
to calling these functions
    if(length(checklist(taxonid = species_id)) > 1){
      # get OBIS records for a given species ID, add year and month, set negative
and missing depth to 0
      obis_recs <- robis::occurrence(
        taxonid = species_id, fields = fields) %>%
        as_tibble()
      if(!"year" %in% names(obis_recs)){
        obis_recs <- obis_recs %>%
          mutate(year = "NA")}
      if(!"month" %in% names(obis_recs)){
        obis_recs <- obis_recs %>%
          mutate(month = NA)}
      obis_recs <- mutate(obis_recs,
                          depth = as.numeric(depth),
                          year = formatC(year),
                          month = formatC(as.numeric(month), width = 2, flag = "0"
),
                          depth0 = case_when(
                            is.na(depth) ~ 0,
                            depth < 0 ~ 0,
                            TRUE ~ depth),
                          valid_AphiaID = species_id)
    } else {
      # at present just returns an empty tibble, which causes problems with other
functions further down the pipeline, hence recommend checking AphiaIDs prior to ca
lling
      obis_recs <- tibble()
    }
  } else {
    obis_recs <- robis::occurrence(
      taxonid = species_id, fields = fields) %>%
      as_tibble()
    if(!"year" %in% names(obis_recs)){
      obis_recs <- obis_recs %>%
        mutate(year = "NA")}
    if(!"month" %in% names(obis_recs)){
      obis_recs <- obis_recs %>%
        mutate(month = NA)}
    obis_recs <- mutate(
      obis_recs,
      depth = as.numeric(depth),
      year = formatC(year),
      month = formatC(as.numeric(month), width = 2, flag = "0"),
      depth0 = case_when(is.na(depth) ~ 0,
      depth < 0 ~ 0,
      TRUE ~ depth),
      valid_AphiaID = species_id
      )
  }
  # return the OBIS records
  obis_recs
}
```

Matching OBIS Records to Bio-ORACLE Temperatures

The next function takes the OBIS records returned above and matches them to Bio-ORACLE SST and SBT. The input is the OBIS records returned above, the other arguments specify the Bio-ORACLE layers to interrogate (`layercodes`) and specify where to save the Bio-ORACLE data (here the assumption is that `use_defaults` is `TRUE`). It returns a tibble with the Bio-ORACLE temperatures added to the OBIS records tibble returned above.

```
get_bio_oracle_t <- function(obis_recs, layercodes = c("BO_sstmean", "BO2_tempmean
_bdmean"),
                             use_defaults = TRUE){
  # Function to match a set of OBIS occurrence recrods to mean SST and SBT from Bi
o-ORACLE
  # Set path for where these two temperature datasets will be stored
  bo_path <- ifelse(use_defaults,
    paste0(file.path(getwd()), "/biooracle"),
    paste0(bespoke_path, "/biooracle"))
  if(!dir.exists(file.path(bo_path))){
  dir.create(path = file.path(bo_path),
    recursive = FALSE, showWarnings = FALSE)}
  # load the layers
  bo_t_dat <- load_layers(layercodes,
    equalarea = TRUE, datadir = "biooracle")
  # Turn the OBIS occurrence locations into spatial points
  points <- SpatialPoints(
    obis_recs[,c("decimalLongitude", "decimalLatitude")],
    lonlatproj)
  # Reproject (could avoid this by setting equalarea = FALSE)
  points <- spTransform(points, equalareaproj)
  # Extract the temperatures for each point
  bo_sst <- raster::extract(bo_t_dat[[1]], points)
  bo_sbt <- raster::extract(bo_t_dat[[2]], points)
  bo_temp <- as_tibble(cbind(bo_sst, bo_sbt))

  # add these temperatures back to the OBIS records and return
  bind_cols(obis_recs, bo_temp)

}
```

Matching OBIS Records to IAP Gridded Temperature

This function takes the OBIS records returned above and matches them to IAP gridded temperature by longitude, latitude, depth, and date. The input is the OBIS records returned above, the other arguments specify where to save the IAP data files (here the assumption is that `use_defaults` is `TRUE`). It returns a tibble with the IAP temperatures added to the OBIS records tibble returned above. NB: this will take a long time to run first time, as each month of climate data will need to be downloaded. Subsequent runs will be much quicker, assuming the path remains the same.

```
get_iap_gridded_t <- function(obis_recs, use_defaults = TRUE, bespoke_path = NULL)
{
  # Function to match a set of OBIS occurrence records to IAP gridded global tempe
rature by month, year, lat, lon, and depth. Returns SST, SBT, and temperature at d
epth.
  # NB: This will take a long time to run first time, as it has to download the cl
imate data. Subsequent runs will be much quicker, assuming the path remains the sa
me
  # Process:
    # 1. get grid cell for occurrence dat
    # 2. group by month, year, deal with occs missing date
    # 3. get relevant IAP data file given month, year
    # 4. match occurrences to temperature by date, lat, lon, depth
    # 5. run over a all months / years

  ################################################
  # Function to run the temperature matching by latitude, longitude, time, and dep
th over all dates
  get_iap_temp_by_lat_lon_time_depth <- function(
    occurrence_dat = obis_recs, fpath = iap_path){

    # group the data by year and month, and get temperature for each record
    matched_temps <- occurrence_dat %>%
      group_by(year, month) %>%
      do(iap_t = get_t_by_grid(., fpath = fpath))
    matched_temps <- unnest(dplyr::select(matched_temps, iap_t))

    matched_temps
    }

  ################################################

  get_gridcell <- function(dll_df,
    depth_vals = c(1, 5, seq(10, 100, by = 10), seq(120, 200, by = 20),
      seq(250, 900, by = 50), seq(1000, 1800, by = 100), 2000),
    lon_vals = 1:360,lat_vals = seq(-89.5, 89.5)){

    # Function to get grid cell index from continuous lat/lon/depth given specifie
d lat/lon/depth grid
    # Defaults here are for a global 1 degree grid, longitude in degrees East, lat
itude in degrees North, with standard depth bands following the World Ocean Atlas
    # Returns a grid index (depth, lon, lat) for one or a vector of locations
    # Accepts a 3-column tibble with cols depth, lon, and lat (in that order, col
names not important)

    # find index for depth, lon and lat
    grid_depth <- findInterval(pull(dll_df[, 1]), depth_vals, all.inside = TRUE)
    grid_lon <- findInterval(pull(dll_df[, 2]), lon_vals, all.inside = TRUE)
    grid_lat <- findInterval(pull(dll_df[, 3]), lat_vals, all.inside = TRUE)
    # add to data frame
    grid_ids <- bind_cols(dll_df,
      grid_depth = grid_depth, grid_lon = grid_lon, grid_lat = grid_lat)
    # return
```

```
    grid_ids
  }

  ################################################

  get_iap_by_month_year <- function(path = iap_path, month = "01", year = "1990")
{
    # Function to get the IAP gridded temperature data for a given month and year
and save it to a specified location
    # build up the file path to search
    fpath <- file.path(path, paste0("CZ16_1_2000m_Temp_year_", year, "_month_", mo
nth, ".nc"))
    # check for existence of required data; get it if it does not exist
    if (!file.exists(fpath)) {
      dir.create(dirname(fpath), recursive = TRUE, showWarnings = FALSE)
      dat_url <- paste0(
      "ftp://ds1.iap.ac.cn/ftp/cheng/CZ16_v3_IAP_Temperature_gridded_1month_netcdf
/Monthly/",
        basename(fpath))
      download.file(dat_url, destfile = fpath)
    }

    # Open file as nc
    iap_t <- nc_open(filename = fpath)
    # get the sea temperature data for this month
    sea_temp <- ncvar_get(iap_t, varid = "temp")
    # close the file connection
    nc_close(iap_t)
    # return the temperature data
    sea_temp
  }

  ################################################

  get_t_by_grid <- function(loc_dat, fpath = iap_path){

    # Function to get temperature by grid cell for a given month and year

    # get month and year to query - assumes data has month, year variables and the
se are the same for all occurrences
    # (NB datasets with multiple months / years are dealt with in subsequent funct
ion)
    loc_m <- loc_dat$month[1]
    loc_y <- loc_dat$year[1]
    st_mat <- get_iap_by_month_year(month = loc_m, year = loc_y, path = fpath)
    st_loc <- st_mat[
      as.matrix(dplyr::select(loc_dat, grid_depth, grid_lon, grid_lat))]
    # sea surface temperature (temperature at depth layer 1)
    sst_loc <- st_mat[
      as.matrix(cbind(1, dplyr::select(loc_dat, grid_lon, grid_lat)))]
    # sea bottom temperature (deepest non-NA temp - will be sea bed in shallow wat
er, t at 2000m in deep)
    # function to find the deepest non-NA temp
    min_na <- function(x){
```

```
      if(sum(is.na(x)) == 0){
        min_na <- length(x) + 1
      } else {
        min_na <- min(which(is.na(x)))
      }
      min_na[min_na > 1] <- min_na[min_na > 1] - 1
      min_na
    }
    # apply this over the full st_mat matrix
    sb_depth <- apply(st_mat, c(2,3), min_na)
    # get sea bottom depth for each observation
    sb_obs <- sb_depth[
      as.matrix(cbind(dplyr::select(loc_dat, grid_lon, grid_lat)))]
    # finally, get the temperature for this combination of depth, lon and lat
    sbt_loc <- st_mat[
      as.matrix(cbind(sb_obs, dplyr::select(loc_dat, grid_lon, grid_lat)))]
    # add the three temperature values back to the locations data, also add sb_dep
th to cross ref with survey depth
    loc_dat <- bind_cols(loc_dat,
      iap_t = st_loc, iap_sst = sst_loc, iap_sbt = sbt_loc,
      iap_grid_bottom_depth = sb_obs)
    loc_dat
    }

  # for later binding - get a unique row id for each record
  obis_recs <- mutate(rowid_to_column(obis_recs, "recordID"),
    lon_deg_east = case_when(
      decimalLongitude >= 0 ~ decimalLongitude,
      decimalLongitude < 0 ~ 360 + decimalLongitude))
  # get grid cell for each depth0, lon and lat
  iap_occs <- obis_recs %>%
    dplyr::select(depth0, lon_deg_east, decimalLatitude) %>%
    get_gridcell()

  iap_occs <- bind_cols(dplyr::select(obis_recs, recordID, year, month), iap_occs)

  # filter out records with no date, or date out of range, or year but no month
  iap_occs <- iap_occs %>%
    mutate(yr = as.numeric(year)) %>%
    filter(!is.na(yr) & yr >= 1940 & yr < 2018 & month != "NA" & !is.na(month))

  if(nrow(iap_occs) > 0){
    # set path
    iap_path <- ifelse(use_defaults, paste0(file.path(getwd()), "/iap_gridded"),
      paste0(bespoke_path, "/iap_gridded"))
    iap_temps <- get_iap_temp_by_lat_lon_time_depth(
      occurrence_dat = iap_occs, fpath = iap_path)
    iap_temps <- left_join(obis_recs,
      dplyr::select(iap_temps, recordID, grid_depth:grid_lat,
        iap_t:iap_grid_bottom_depth), by = c("recordID"))
  } else {
    iap_temps <- mutate(obis_recs,
      grid_depth = NA, grid_lon = NA, grid_lat = NA,
      iap_t = NA, iap_sst = NA, iap_sbt = NA, iap_grid_bottom_depth = NA)
```

```
  }

  # Tidy up data
  iap_temps <- dplyr::select(iap_temps, -recordID, -lon_deg_east)

  # Return
  iap_temps
}
```

Saving Temperature-Matched OBIS Records to File

This function simply saves the temperature-matched OBIS records to file. It uses a sensible path and creates a filename based on the species' Aphia ID, and the date.

```
save_full_recs <- function(rec_df, save_recs = TRUE, use_defaults = TRUE, bespoke_
path = NULL){
  # if save_recs == TRUE, save the full set of obis records + IAP + BO temperature
for a species

  if(save_recs == TRUE){
    out_path <- ifelse(use_defaults, paste0(file.path(getwd()),
      "/t_matched_obis_recs"),
    paste0(bespoke_path, "/t_matched_obis_recs"))

  if(!dir.exists(file.path(out_path))){
    dir.create(path = file.path(out_path), recursive = FALSE, showWarnings = FALSE
)}

  # paste together the filename
  sp_filename <- paste0("aphia", rec_df$valid_AphiaID[1],
    "_obis_iap_bo_", Sys.Date(), ".csv")

  # write the file
  write_csv(x = rec_df, path = file.path(paste(out_path, sp_filename, sep = "/")))
  }

  # Return the (unchanged) data to pass to next function
  rec_df
}
```

Summarising the Temperature Affinity of a Species

This final function takes the record-level data and creates a species-level summary, returning a range of summary statistics for the species.

```
t_summary <- function(t_matched_dat){
  # Function to get a range of temperature summary stats from a matched obis-IAP-b
io-oracle data frame
  counts <- summarise(t_matched_dat, n_obis_rec = n())
  missings <- miss_var_summary(dplyr::select(
    t_matched_dat, iap_t:iap_sbt, bo_sst:bo_sbt))
  missings_df <- t(missings[, "n_miss"])
  colnames(missings_df) <- paste0(pull(missings, variable), "_NA")
  missings_df <- as_tibble(missings_df)
  # define separate functions for 5% and 95% quantiles
  q5 <- function(x, na.rm = TRUE){stats::quantile(x, 0.05, na.rm = TRUE)}
  q95 <- function(x, na.rm = TRUE){stats::quantile(x, 0.95, na.rm = TRUE)}

  # get a range of summary stats over all variables in the dataset
  t_stats <- summarise_at(t_matched_dat,
    vars(iap_t:iap_sbt, bo_sst:bo_sbt),
    tibble::lst(mean, min, max, median, sd, mad, q5, q95), na.rm = TRUE)

  # Tidy up and return the species-level summary
  t_summ <- bind_cols(counts, missings_df, t_stats)

  t_summ
}
```

An example with one species

This is an example of how to run the above code for a single species - we use *Scytothamnus fasciculatus*, Aphia ID 325567, chosen as it has just 6 OBIS records so should run reasonably quickly.

```
s_fasc_t_affin <- get_temp_summ_by_sp(sp_id = 325567)
```

Processing Fish Species from the Comte-Olden Dataset

This shows how to run the temperature matching functions on the marine fish species included in the Comte-Olden thermal limits data set (see Comte & Olden 2017, https://doi.org/10.1038/nclimate3382 (https://doi.org/10.1038/nclimate3382)). The data are available to download via figshare here https://figshare.com/s/eca5d4c047e8c87172db (https://figshare.com/s/eca5d4c047e8c87172db) - we downloaded the spreadsheet `Original_data` and saved it (unchanged) as a CSV.

Reading and Formatting the Data

The CSV data needs to be read in and some filtering and formatting performed. Parsing failures are not relevant to the variables used in our analyses.

```
co_exp_dat <- read_csv("data/Comte__Olden_Data.csv")
```

This dataset needs to be restricted to marine species, and we then get summaries by species (number of

studies, mean, min, and max reported CTmax, weighted mean (weighted by 1/SD of the individual estimates), and a single `CT_max` estimate which is the weighted mean if available, simple arithmetic mean if not (e.g. if no SDs were available))

```
co_exp_dat <- co_exp_dat %>%
  filter(`Realm affinity` == "Marine") %>%
  group_by(Species, Family) %>%
  summarise(
    n_studies = n(),
    mean_CT_max = mean(`Thermal limit (°C)`),
    min_CT_max = min(`Thermal limit (°C)`),
    max_CT_max = max(`Thermal limit (°C)`),
    wt_mean_CT_max = weighted.mean(`Thermal limit (°C)`, w = 1/`SD Thermal limit`)
  ) %>%
  mutate(CT_max = ifelse(is.na(wt_mean_CT_max), mean_CT_max, wt_mean_CT_max))
```

The next step is get a WoRMS Aphia ID for all species, using the `worrms` package to interface to the World Register of Marine Species (http://www.marinespecies.org (http://www.marinespecies.org)) and add it back into the data frame. This may take a minute or two to run over all species:

```
aphias <- wm_name2id_(co_exp_dat$Species)
co_exp_dat <- bind_cols(co_exp_dat, aphiaID = unlist(aphias))
```

Some species return -99 as an Aphia ID:

```
filter(co_exp_dat, aphiaID < 0) %>% dplyr::select(Species, Family)
```

```
## # A tibble: 4 x 2
## # Groups:   Species [4]
##   Species               Family
##   <chr>                 <chr>
## 1 Plotosus lineatus     Plotosidae
## 2 Pterois volitans      Scorpaenidae
## 3 Scolopsis taenioptera Nemipteridae
## 4 Symphodus ocellatus   Labridae
```

Check these names on marinespecies.org and add in the correct Aphia ID:

```
co_exp_dat <- co_exp_dat %>%
  mutate(aphiaID = case_when(
    Species == "Plotosus lineatus" ~ as.integer(217659),
    Species == "Pterois volitans" ~ as.integer(159559),
    Species == "Scolopsis taenioptera" ~ as.integer(276789),
    Species == "Symphodus ocellatus" ~ as.integer(273572),
    TRUE ~ aphiaID
  ))
```

The next step is to get the *valid* Aphia ID associated with all of these returned IDs:

```
aphia_worms <- wm_record_(id = co_exp_dat$aphiaID)
valid_aphias <- unlist(lapply(aphia_worms, "[[", "valid_AphiaID"))
co_exp_dat <- bind_cols(co_exp_dat, valid_AphiaID = as.vector(valid_aphias))
```

To classify fish into broad habitat / functional groups, we use the DemersPelag field from FishBase, accessed via `rfishbase` , and add them back into the dataset:

```
spp_depths <-  species(pull(co_exp_dat, Species),
  fields = c("Species", "DemersPelag", "DepthRangeShallow", "DepthRangeDeep"))
co_exp_dat <- left_join(co_exp_dat, spp_depths, by = "Species")
```

NB 3 species are missing habitat clasifications:

```
co_exp_dat %>% filter(is.na(DemersPelag)) %>% dplyr::select(Species, Family, Demer
sPelag)
```

```
## # A tibble: 3 x 3
## # Groups:   Species [3]
##   Species               Family    DemersPelag
##   <chr>                 <chr>     <chr>
## 1 Chelon subviridis     Mugilidae <NA>
## 2 Diplodus sargus sargus Sparidae  <NA>
## 3 Moolgarda engeli      Mugilidae <NA>
```

Checking on Fishbase, *Chelon subviridis* is listes as demersal, *Diplodus sargus sargus* is included as *Diplodus sargus* which is demersal, *Moolgarda engeli* is listed as reef-associated. Add this info:

```
co_exp_dat <- co_exp_dat %>% mutate(
  DemersPelag = case_when(
    Species == "Chelon subviridis" ~ "demersal",
    Species == "Diplodus sargus sargus" ~ "demersal",
    Species == "Moolgarda engeli" ~ "reef-associated",
    TRUE ~ DemersPelag
  )
)
```

Some habitat categories have low numbers of species:

```
co_exp_dat %>% count(DemersPelag)
```

```
## # A tibble: 158 x 3
## # Groups:   Species [158]
##    Species                      DemersPelag          n
##    <chr>                        <chr>            <int>
##  1 Abudefduf saxatilis          reef-associated      1
##  2 Abudefduf sordidus           reef-associated      1
##  3 Abudefduf troschelii         reef-associated      1
##  4 Acanthemblemaria hancocki    reef-associated      1
##  5 Acanthoclinus littoreus      demersal             1
##  6 Acanthopagrus latus          demersal             1
##  7 Achirus mazatlanus           demersal             1
##  8 Albula vulpes                reef-associated      1
##  9 Ambassis kopsii              demersal             1
## 10 Amblygobius phalaena         reef-associated      1
## # … with 148 more rows
```

So we aggregate into a new `habitat` variable:

```
co_exp_dat <- co_exp_dat %>%
  mutate(habitat = case_when(
    DemersPelag %in% c("bathydemersal", "demersal") ~ "demersal",
    DemersPelag %in% c("pelagic-neritic", "pelagic-oceanic") ~ "pelagic",
    TRUE ~ DemersPelag
  ))
```

Now we get a summary of OBIS records for all species, accessing https://obis.org (https://obis.org) via the `robis` package, filtering the output to remove any duplicates (e.g. taxa present in OBIS as both species and subspecies with the same valid Aphia ID):

```
aphia_checklist_exp <- co_exp_dat %>%
  ungroup() %>%
  group_by(valid_AphiaID) %>%
  do(checklist(taxonid = .$valid_AphiaID)) %>%
  ungroup() %>%
  filter(!duplicated(valid_AphiaID))
```

This shows that there are records for 157 of the 158 species in the data set, and 2639684 OBIS records in total for these species. Check taxonomic distribution of the species:

```
aphia_checklist_exp %>% count(order)
```

```
## # A tibble: 17 x 2
##    order                  n
##    <chr>              <int>
##  1 Albuliformes           1
##  2 Anguilliformes         1
##  3 Atheriniformes         2
##  4 Beloniformes           2
##  5 Clupeiformes           5
##  6 Cyprinodontiformes     2
##  7 Gadiformes             1
##  8 Gobiesociformes        3
##  9 Gonorhynchiformes      1
## 10 Myliobatiformes        1
## 11 Perciformes          117
## 12 Pleuronectiformes     11
## 13 Salmoniformes          1
## 14 Scorpaeniformes        5
## 15 Siluriformes           1
## 16 Syngnathiformes        1
## 17 Tetraodontiformes      2
```

Join relevant info back to `co_exp_dat`, ungroup, and remove species with no OBIS records:

```
co_exp_dat <- co_exp_dat %>%
  left_join(dplyr::select(aphia_checklist_exp, valid_AphiaID, class:order, records
),
            by = "valid_AphiaID") %>%
  ungroup() %>%
  filter(!is.na(records))
```

This dataset is now ready to run the temperature matching routines outlined below.

Processing Marine Species from the GlobTherm Database

This section formats thermal tolerance data taken from the GlobTherm Database, described in Bennett et al. (2018a, https://doi.org/10.1038/sdata.2018.22 (https://doi.org/10.1038/sdata.2018.22)) with the data itself published in Dryad (Bennett et al. 2018b, https://doi.org/10.5061/dryad.1cv08 (https://doi.org/10.5061/dryad.1cv08)). We downloaded the file in CSV format from Dryad, `GlobalTherm_upload_02_11_17.csv`, which is then read in and formatted as follows (again any warnings and parsing failures do not affect the data we need):

```
globtherm_dat <- read_csv("data/GlobalTherm_upload_02_11_17.csv")
```

Some species have two records of `Tmax` (upper temperature limit), these can be examined using:

```
globtherm_dat %>%
  mutate(sciname = paste(Genus, Species)) %>%
  dplyr::select(sciname, Tmax, max_metric, Tmax_2, max_metric_2) %>%
  filter(!is.na(Tmax_2))
```

```
## # A tibble: 207 x 5
##    sciname                            Tmax max_metric Tmax_2 max_metric_2
##    <chr>                             <dbl> <chr>       <dbl> <chr>
##  1 Bangia atropurpurea                23   LT0            24 LT100
##  2 Bangia fuscopurpurea               25   LT0            28 LT100
##  3 Porphyra linearis                  23   LT0            25 LT100
##  4 Porphyra umbilicalis               24.3 LT0            28 LT100
##  5 Smithora naiadum                   20   LT0            23 LT100
##  6 Antithamnion furcellatum           30   LT0            35 LT100
##  7 "Antithamnion sarniense\xa0"       27   LT0            30 LT100
##  8 Asparagopsis armata                27   LT0            30 LT100
##  9 Callophyllis laciniata             24   LT0            27 LT100
## 10 Dilsea carnosa                     30   LT0            35 LT100
## # … with 197 more rows
```

All of these report both LT0 and LT100 - this is consistent with the meta data provided by Bennett et al: >Tmax_2 is only for algae: upper lethal limit with 100% mortality (in degrees °C); max_metric_2 Only for algae, always LT100 (100% death); max_interval_before_LT100 Only in the case of algae. The size of the interval between experimental water baths in °C directly before all individuals were recorded as dead (LT100). i.e. an LT100 of 10°C with an interval of 10°C would indicate that the all specimens were dead in a water bath of 10°C and that the previous recording was taken in a water bath at 10°C cooler (0°C) in which some individuals were recorded as alive.

So here we use `Tmax_2` as the upper temperature limit in all cases when it is recorded. The dataset is tidied as follows:

```
globtherm_dat <- globtherm_dat %>%
  mutate(sciname = paste(Genus, Species),
    T_max = case_when(!is.na(Tmax_2) ~ Tmax_2, TRUE ~ Tmax),
    T_metric = case_when(!is.na(Tmax_2) ~ max_metric_2, TRUE ~ max_metric)
  ) %>%
  dplyr::select(
    sciname, N, T_max, T_metric, error, `error measure`, `Quality of UTNZ`, REF_ma
x)
```

We use WoRMS to identify marine species. This requires a small function building on the `worrms` package to deal with species names not in WoRMS and to identify different kinds of errors:

```
get_wormsID <- function(sp_name){
  wm_id <- try(wm_name2id(sp_name), silent = TRUE)
  if(class(wm_id) == "try-error"){
    wm_id <- wm_id %>% attr("condition") %>% as.character() %>% parse_number()
    wm_id <- -1*wm_id
  }
  tibble(sciname = sp_name, aphiaID = unlist(wm_id))
}
```

This is then run over all species in GlobTherm (takes a few minutes):

```
aphias <- globtherm_dat %>%
  group_by(sciname) %>%
  do(get_wormsID(sp_name = .$sciname)) %>%
  ungroup()
```

There are two error codes that this function returns (both converted to negatives to avoid any confusion with valid AphiaIDs), -204 is derived from `Error: (204) No Content` and indicates no match with any record in WoRMS, -206 is derived from `simpleError: (206) Partial Content` which indicates a likely record in WoRMS that should be investigated. In total, positive AphiaIDs were returned for 586 species with a further 27 species having the partial content error code. These are filled in following a direct search of marinespecies.org:

```
aphias <- aphias %>% mutate(aphiaID = case_when(
  sciname == "Achillea millefolium" ~ 993723,
  sciname == "Bangia fuscopurpurea" ~ 157261,
  sciname == "Caloglossa leprieurii" ~ 214170,
  sciname == "Celmisia brevifolia" ~ 1090682,
  sciname == "Ceramium deslongchampsii" ~ 144545,
  sciname == "Cladophora rupestris" ~ 145064,
  sciname == "Colpomenia peregrina" ~ 145856,
  sciname == "Cookia sulcata" ~ 413403,
  sciname == "Dictyota dichotoma" ~ 145367,
  sciname == "Dictyota divaricata" ~ 658996,
  sciname == "Euryops virgineus" ~ 1098760,
  sciname == "Felicia amelloides" ~ 1085001,
  sciname == "Gobius niger" ~ 126892,
  sciname == "Hypoglossum hypoglossoides" ~ 144756,
  sciname == "Laurencia intricata" ~ 144823,
  sciname == "Littorina saxatilis" ~ 140264,
  sciname == "Notemigonus crysoleucas" ~ 159989,
  sciname == "Paracalliope fluviatilis" ~ 548050,
  sciname == "Petrolisthes gracilis" ~ 493072,
  sciname == "Phyllodictyon anastomosans" ~ 146242,
  sciname == "Polysiphonia elongata" ~ 144628,
  sciname == "Porphyra umbilicalis" ~ 144437,
  sciname == "Rhinichthys atratulus" ~ 159985,
  sciname == "Striaria attenuata" ~ 548021,
  sciname == "Succinea strigata" ~ 1324835,
  sciname == "Ulva fasciata" ~ 145982,
  sciname == "Xantho incisus" ~ 148461,
  TRUE ~ aphiaID))
```

The next job is to get OBIS summary information for all species (filtering out subspecies which are duplicate IDs) - NB `checklist` automatically associates invalid aphiaIDs with the appropriate valid ID, and should filter out most non-marine species which have aphia IDs as they will not be recorded in OBIS. It will also return the `is_marine` flag from WoRMS to allow any other non-marine species to be eliminated at this point. However it can fail if fed valid Aphia IDs from non-marine taxa, so we need to catch these errors in a slightly modified function:

```
checklist_catcherror <- function(taxonid){
  cl <- try(checklist(taxonid = taxonid), silent = TRUE)
  if(identical(class(cl), "try-error")){cl <- tibble()}
  cl
}
```

Which can then be run over all Aphia IDs:

```
aphia_checklist <- aphias %>%
  filter(aphiaID > 0) %>%
  group_by(aphiaID) %>%
  do(checklist_catcherror(taxonid = .$aphiaID)) %>%
  ungroup() %>%
  filter(!duplicated(taxonID) & is_marine == TRUE)
```

This shows that we have OBIS records for 478 of 613 species for which we have aphiaIDs and 3243403 records in total. Add back in the original GlobTherm scientific names, and filter to Species rank:

```
aphia_checklist <- aphia_checklist %>%
  left_join(aphias, by = "aphiaID") %>%
  dplyr::select(sciname, everything()) %>%
  filter(taxonRank == "Species")
```

Join relevant columns back into `globtherm_dat`, and filter to species with an Aphia ID:

```
globtherm_dat <- globtherm_dat %>%
  left_join(dplyr::select(aphia_checklist, sciname, taxonID, acceptedNameUsage,
                          phylum, class, order, family, records),
            by = "sciname") %>%
  rename(valid_AphiaID = taxonID, scientificName = acceptedNameUsage) %>%
  dplyr::select(valid_AphiaID, everything()) %>%
  filter(!is.na(valid_AphiaID))
```

At this point you can check the taxonomic distribution of species if required, e.g.

```
globtherm_dat %>% count(phylum) %>% arrange(desc(n))
```

```
## # A tibble: 11 x 2
##    phylum            n
##    <chr>         <int>
##  1 Rhodophyta      114
##  2 Chordata         99
##  3 Ochrophyta       92
##  4 Mollusca         43
##  5 Arthropoda       33
##  6 Chlorophyta      28
##  7 Echinodermata     6
##  8 Annelida          2
##  9 Brachiopoda       2
## 10 Bryozoa           1
## 11 Tracheophyta      1
```

Check if there are any duplicate valid AphiaIDs:

```
sum(duplicated(globtherm_dat$valid_AphiaID))
```

```
## [1] 0
```

Adding Functional Group Information to GlobTherm Species

We used the attributes data provided by WoRMS to add functional group to the GlobTherm species. This uses the following function (available from https://github.com/tomjwebb/WoRMS-functional-groups (https://github.com/tomjwebb/WoRMS-functional-groups)), building on the `worrms` package:

```
get_worms_fgrp <- function(AphiaID){
  # Function to get functional group info for an Aphia ID

  # First, test if the species has attribute data
  attr_dat <- try(wm_attr_data(
    AphiaID, include_inherited = TRUE), silent = TRUE)
  if(!identical(class(attr_dat), "try-error")){
  # if attribute data exists, test if functional group is there
    if("Functional group" %in% attr_dat$measurementType){
      fg_dat <- attr_dat %>%
        filter(measurementType == "Functional group")
      # assign the $children - so that it can be used
      children <- data.frame(fg_dat$children)
      if(length(children) > 0 ){
        # Extract the life stage information from the $children field
        life_stage <- bind_rows(fg_dat$children) %>%
        dplyr::select(measurementValue) %>%
          rename(stage = measurementValue) %>%
          bind_cols(., fg_dat)
        # create the output to return
        out <- tibble(
```

```
          AphiaID = as.numeric(life_stage$AphiaID),
          stage = life_stage$stage,
          fun_grp = life_stage$measurementValue)
      } else {
        # If no life stage info, assume stage is adult
        out <- tibble(
          AphiaID = as.numeric(fg_dat$AphiaID),
          stage = "adult",
          fun_grp = fg_dat$measurementValue)
      }
    } else  if ("Paraphyletic group" %in% attr_dat$measurementType) {
      # get paraphyletic group info if available
      out <- tibble(
        AphiaID = AphiaID, stage = "adult",
        fun_grp = first(attr_dat$measurementValue[
          attr_dat$measurementType == "Paraphyletic group"])
      )
    } else {
      # check taxonomy for other groups
      taxo_dat <- wm_classification(AphiaID)
      fg <- case_when(
        "Aves" %in% taxo_dat$scientificname ~ "birds",
        "Mammalia" %in% taxo_dat$scientificname ~ "mammals",
        "Reptilia" %in% taxo_dat$scientificname ~ "reptiles",
        TRUE ~ as.character(NA)
      )
      out <- tibble(AphiaID = AphiaID, stage = "adult",
        fun_grp = fg)
    }
  } else {
    # check taxonomy for other groups
    taxo_dat <- wm_classification(AphiaID)
    fg <- case_when(
      "Aves" %in% taxo_dat$scientificname ~ "birds",
      "Mammalia" %in% taxo_dat$scientificname ~ "mammals",
      "Reptilia" %in% taxo_dat$scientificname ~ "reptiles",
      TRUE ~ as.character(NA)
    )
    out <- tibble(AphiaID = AphiaID, stage = "adult",
      fun_grp = fg)
  }
  # output the fg data
  out
}
```

This can be run for a single taxon, e.g.

```
get_worms_fgrp(AphiaID = 100803)
```

```
## # A tibble: 2 x 3
##   AphiaID stage           fun_grp
##     <dbl> <chr>           <chr>
## 1  100803 adult           benthos
## 2  100803 larva > planula plankton > zooplankton
```

But to run for all species in the GlobTherm data and tidy the output (adding functional group for each life stage as a separate variable), use:

```
globtherm_fgrp <- dplyr::select(globtherm_dat, valid_AphiaID) %>%
  group_by(valid_AphiaID) %>%
  do(get_worms_fgrp(AphiaID = .$valid_AphiaID)) %>%
  mutate(functional_group = case_when(
    str_detect(fun_grp, ">") ~ tolower(word(fun_grp, -1)),
    fun_grp == "Pisces" ~ "fish",
    TRUE ~ fun_grp
    )) %>%
  dplyr::select(-c(fun_grp, AphiaID)) %>%
  spread(stage, functional_group)
```

Rename based on stages present:

```
globtherm_fgrp <- globtherm_fgrp %>%
  rename(fun_grp = adult,
    fun_grp_larva = larva,
    fun_grp_nauplius = nauplius)
```

This gets a group for almost all (411/ 421) species. Join the results back to globtherm_dat:

```
globtherm_dat <- left_join(globtherm_dat, globtherm_fgrp,
  by = "valid_AphiaID")
```

Check for species missing functional group info:

```
missing_sp <- globtherm_dat %>%
  filter(is.na(fun_grp)) %>%
  dplyr::select(valid_AphiaID, scientificName, phylum:class, fun_grp)
```

Check for info on these from SeaLifeBase using `rfishbase` :

```
missing_sp_slb <- pull(missing_sp, scientificName) %>%
  species(server = "https://fishbase.ropensci.org/sealifebase") %>%
  dplyr::select(Species, DemersPelag)
```

Add to `missing_sp` :

```
missing_sp <- missing_sp %>%
  left_join(dplyr::select(missing_sp_slb, Species, DemersPelag),
  by = c("scientificName" = "Species"))
```

Standardise functional groups and add missing values. Sources and justifications for ambiguous or

unknown functional groups are: *Orchomenella pinguides* is a deposit-feeding benthic amphipod (Sfiligoj et al. 2015, Ecotoxicology (2015) 24: 583, https://doi.org/10.1007/s10646-014-1406-4 (https://doi.org/10.1007/s10646-014-1406-4)); *Onisimus litoralis* is sediment dwelling in intertidal and shallow subtidal, http://www.arcodiv.org/seaice/amphipods/Onisimus_litoralis.html (http://www.arcodiv.org/seaice/amphipods/Onisimus_litoralis.html); *Paracalliope australis* lives in tide pools grazing micro-algae from the rocks and from the faecal pellet layer (McGrowther 1983 Australian Journal of Marine and Freshwater Research 34(5) 717 - 726, https://doi.org/10.1071/MF9830717 (https://doi.org/10.1071/MF9830717)) and so is considered benthic; all Rhodophyta are considered to be macroalgae, and for our purposes we also class the surf grass *Phyllospadix scouleri* (http://www.marinespecies.org/aphia.php?p=taxdetails&id=374707#attributes (http://www.marinespecies.org/aphia.php?p=taxdetails&id=374707#attributes)) as macroalgae:

```
missing_sp <- missing_sp %>%
  mutate(fun_grp = case_when(
    phylum  == "Rhodophyta" | phylum == "Tracheophyta" ~ "macroalgae",
    phylum == "Arthropoda" ~ "benthos"
    ))
```

Add this info back to globtherm_dat:

```
globtherm_dat <- globtherm_dat %>%
  mutate(fun_grp = case_when(
    phylum  == "Rhodophyta" | phylum == "Tracheophyta" ~ "macroalgae",
    is.na(fun_grp) ~ "benthos",
    TRUE ~ fun_grp
  ))
```

NB. There are still 29 species listed simply as ‘Algae’. Examine the taxonomic makeup of these:

```
globtherm_dat %>% filter(fun_grp == "Algae") %>% count(class)
```

```
## # A tibble: 2 x 2
##   class            n
##   <chr>        <int>
## 1 Phaeophyceae    24
## 2 Ulvophyceae      5
```

All of the Phaeophyceae are macroalgae. The Ulvophyceae includes both macroalgae and unicellular algae, so examine a little further:

```
globtherm_dat %>% filter(class == "Ulvophyceae" & fun_grp == "Algae") %>% dplyr::s
elect(scientificName, family)
```

```
## # A tibble: 5 x 2
##   scientificName                 family
##   <chr>                          <chr>
## 1 Lychaete pellucida             Cladophoraceae
## 2 Pseudorhizoclonium africanum Cladophoraceae
## 3 Acrosiphonia arcta             Ulotrichaceae
## 4 Acrosiphonia sonderi           Ulotrichaceae
## 5 Urospora penicilliformis       Ulotrichaceae
```

All of these are macroalgae, so:

```
globtherm_dat <- globtherm_dat %>% mutate(
  fun_grp = ifelse(fun_grp == "Algae", "macroalgae", fun_grp)
  )
```

Running the Temperature Matching Functions on Comte-Olden Species

This code runs the full temperature-matching process over all species in the Comte-Olden dataset. **WARNING**: the first time this is run it will take **MANY HOURS**. It will also download a ~10MB file for every unique month of data which has occurrence records, so you need to ensure you have space for this. We have developed a parallelised workflow that makes this more efficient for large species lists, if you have access to a cluster - see https://github.com/EMODnet/EMODnet-Biology-thermal-traits (https://github.com/EMODnet/EMODnet-Biology-thermal-traits) - but here we work on a single core on a standalone machine. Subsequent runs in the same directory will be very quick, if `check_match` is set to `TRUE`. It's a good idea to try running the code on a small subset of species if you want to try it out - but NB even this is likely to take over an hour first time you run it, depending how many individual months of IAP gridded data need to be downloaded:

```
samp_species_t <- co_exp_dat %>%
  slice(2:4) %>%
  group_by(valid_AphiaID) %>%
  do(get_temp_summ_by_sp(sp_id = .$valid_AphiaID,
    save_all_recs = TRUE, check_match = TRUE, use_defaults = TRUE))
```

To run over the whole list of species from the Comte-Olden dataset - same **WARNING** as above:

```
co_species_t <- co_exp_dat %>%
  group_by(valid_AphiaID) %>%
  do(get_temp_summ_by_sp(sp_id = .$valid_AphiaID,
                         save_all_recs = TRUE, check_match = TRUE,
                         use_defaults = TRUE)) %>%
  ungroup()
```

The temperature affinities can then be added back into `co_exp_dat`:

```
co_exp_dat <- co_exp_dat %>%
  left_join(dplyr::select(co_species_t, - species_id), by = "valid_AphiaID")
```

We can use the species habitat descriptions to assign an ‘optimal’ temperature to each species from the Bio-ORACLE temperature data: bottom temperature for bottom dwellers, surface temperature for pelagics. We use surface temperature for reef-associated species too as they are typically rather shallow-dwelling and SST is more widely available.

```
co_exp_dat <- co_exp_dat %>%
  mutate(
    bo_t_opt = case_when(
      DemersPelag %in% c("benthopelagic", "demersal", "bathydemersal") ~ bo_sbt_me
an,
      DemersPelag %in% c("reef-associated", "pelagic-neritic", "pelagic-oceanic")
~ bo_sst_mean),
    bo_t_opt_sd = case_when(
      DemersPelag %in% c("benthopelagic", "demersal", "bathydemersal") ~ bo_sbt_sd
,
      DemersPelag %in% c("reef-associated", "pelagic-neritic", "pelagic-oceanic")
~ bo_sst_sd),
    bo_t_opt95 = case_when(
      DemersPelag %in% c("benthopelagic", "demersal", "bathydemersal") ~ bo_sbt_q9
5,
      DemersPelag %in% c("reef-associated", "pelagic-neritic", "pelagic-oceanic")
~ bo_sst_q95)
  )
```

Shortcut: read in T-matched Comte-Olden data

This is now the full marine C-O dataset with temperature affinity summaries for all species, which is the same data set as was read in directly at the top of this document, fully formatted and ready for the figures and analyses:

```
co_exp_dat <- read_csv("data/t_matched_co_exp_dat_full.csv")
```

Running the Temperature Matching Functions on GlobTherm Species

Now run the temperature matching functions as for the Comte-Olden species. NB - as before, this will take **MANY HOURS** to run the first time (and you may wish to explore our parallelised version https://github.com/EMODnet/EMODnet-Biology-thermal-traits (https://github.com/EMODnet/EMODnet-Biology-thermal-traits) for large species lists), but running again in the same directory with `check_match = TRUE` will be very much quicker (a few minutes at most).

```
globtherm_t <- globtherm_dat %>%
  group_by(valid_AphiaID) %>%
  do(get_temp_summ_by_sp(sp_id = .$valid_AphiaID,
                         save_all_recs = TRUE, check_match = TRUE,
                         use_defaults = TRUE)) %>%
  ungroup()
```

Join this back to `globtherm_dat`

```
globtherm_dat <- globtherm_dat %>% left_join(globtherm_t,
  by = "valid_AphiaID")
```

Get 'optimal' BioORACLE temperature data based on habitat / group - bottom temperature for bottom dwellers, surface temperature for pelagics. First, further classify fish into demersal / pelagic using FishBase:

```
spp_depths <- globtherm_dat %>%
  filter(fun_grp == "fish") %>%
  pull(scientificName) %>%
  species(fields=c("Species", "DemersPelag", "DepthRangeShallow", "DepthRangeDeep"
))
```

Join back to `globtherm_dat` :

```
globtherm_dat <- globtherm_dat %>%
  left_join(dplyr::select(spp_depths, Species:DemersPelag),
  by = c("scientificName" = "Species"))
```

Use SST for pelagic fish, birds, mammals, SBT for demersal fish, benthos, macroalgae

```
globtherm_dat <- globtherm_dat %>%
  mutate(
    bo_t_opt = case_when(
      DemersPelag %in%
        c("benthopelagic", "demersal", "bathydemersal") ~ bo_sbt_mean,
      DemersPelag %in%
        c("reef-associated", "pelagic-neritic", "pelagic-oceanic") ~ bo_sst_mean,
      fun_grp %in% c("benthos", "macroalgae") ~ bo_sbt_mean,
      fun_grp %in% c("birds", "mammals", "nekton") ~ bo_sst_mean
    ),
    bo_t_opt_sd = case_when(
      DemersPelag %in%
        c("benthopelagic", "demersal", "bathydemersal") ~ bo_sbt_sd,
      DemersPelag %in%
        c("reef-associated", "pelagic-neritic", "pelagic-oceanic") ~ bo_sst_sd,
      fun_grp %in% c("benthos", "macroalgae") ~ bo_sbt_sd,
      fun_grp %in% c("birds", "mammals", "nekton") ~ bo_sst_sd
    ),
    bo_t_opt95 = case_when(
      DemersPelag %in%
        c("benthopelagic", "demersal", "bathydemersal") ~ bo_sbt_q95,
      DemersPelag %in%
        c("reef-associated", "pelagic-neritic", "pelagic-oceanic") ~ bo_sst_q95,
      fun_grp %in% c("benthos", "macroalgae") ~ bo_sbt_q95,
      fun_grp %in% c("birds", "mammals", "nekton") ~ bo_sst_q95
    )
  )
```

Shortcut: read in T-matched Globtherm data

This is now the full marine Globtherm dataset with temperature affinity summaries for all species, as read in as a shortcut at the top of this document. This is provided as a csv file which can be directly read in here, circumventing the above:

```
globtherm_dat <- read_csv("data/t_matched_globtherm_dat_full.csv")
```

Analysis and Visualisation

Species Occurring in Both Datasets

To check results from species found in both Comte-Olden and GlobTherm, first create a combined dataset:

```
comb_dat <- inner_join(
  dplyr::select(co_exp_dat, valid_AphiaID,
    mean_CT_max:CT_max, bo_sst_mean),
  dplyr::select(globtherm_dat, valid_AphiaID,
    sciname, T_max, bo_sst_mean, records),
  by = "valid_AphiaID")
```

Check the correlation between thermal limits across the two datasets for these 45 species, first plotting T_{max} v CT_{max} with 1:1 line:

```
(t_co_v_globtherm <- ggplot(
  comb_dat, aes(x = CT_max, y = T_max)) +
  geom_point() +
  geom_abline(slope = 1, intercept = 0, linetype = "dashed") +
  xlab(expression(paste(CT[max], " (Comte-Olden)"))) +
  ylab(expression(paste(T[max], "(GlobTherm)")))
)
```

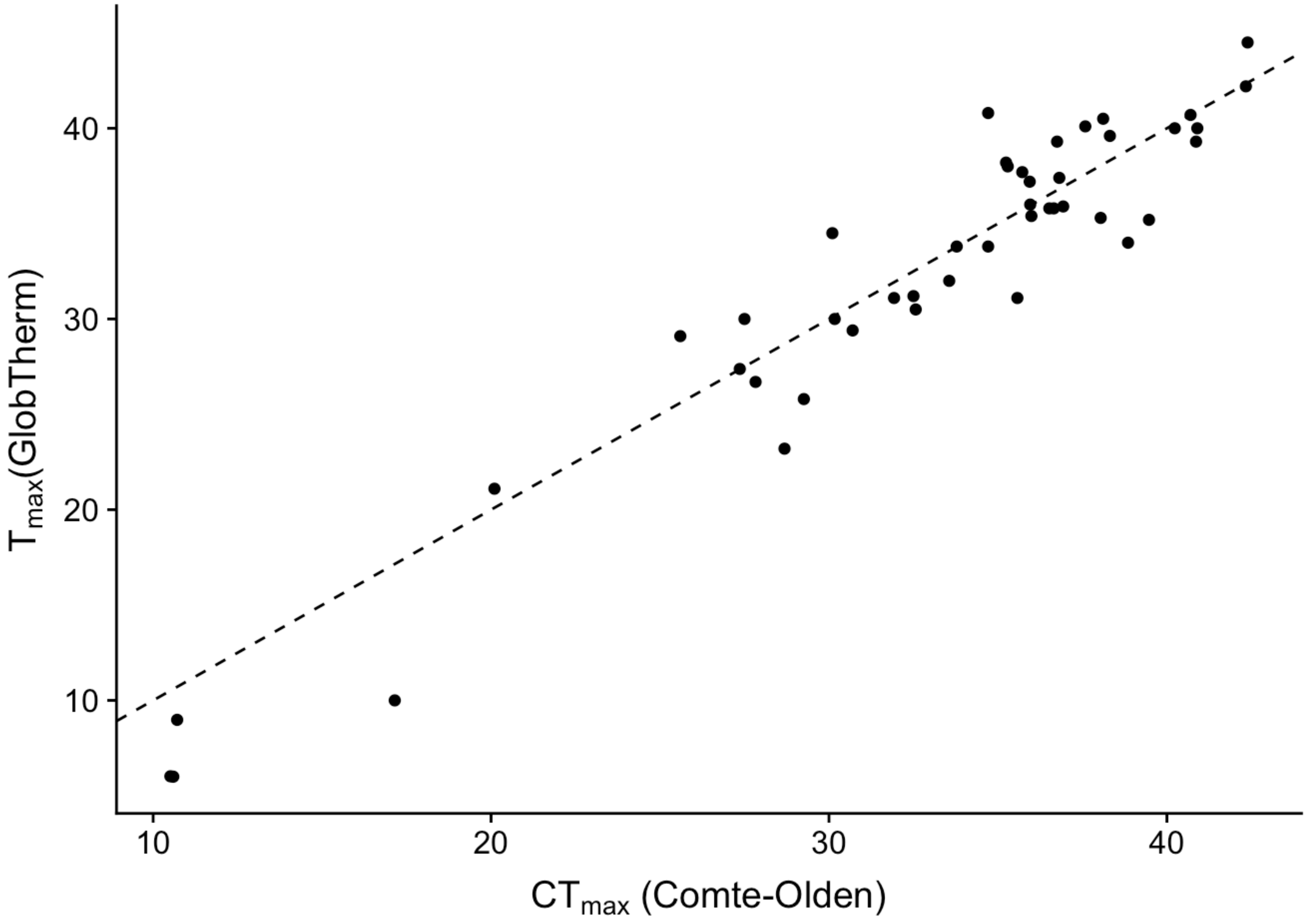

Figure S1. Relationships between experimentally-derived upper thermal limits from both data sources (Comte-Olden and Globtherm) for the 45 marine species occurring in both datasets. The correlation is 0.959. The dashed line indicates a 1:1 relationship.

The correlation is very strongly positive (r = 0.959), and the regression coefficient (1.109) is very close to 1 (95%CI 1.008-1.21) - this shows that, despite differences in methodology and inclusion criteria, thermal limits are very similar within species across the two datasets. However, there are some variations so we consider these datasets separately for most of our analyses.

Summary of OBIS Occurrence records for included species

This code reads in all the t-matched records as a single data frame. NB - this requires all of the (temperature-matched) individual occurrence records for each species, each species' occurrences stored in a separate file. The above code will generate these but we do not provide them here, so the following section of code will only run if you have run the full t-matching code. We do provide summary data that will generate the map though, see the Map Occurrences section. First, get a list of all the processed files (this assumes you saved all the occurrence records when running the t-matching functions, and used the default file structure):

```
files <- list.files(path = "./t_matched_obis_recs", pattern = "*.csv")
```

Extract just the aphia IDs from these filenames:

```
done_aphias <- files %>%
    word(sep = "_") %>%
    str_replace(pattern = "aphia", replacement = "") %>%
    as.integer()
```

Remove any duplicates or NA values:

```
done_aphias <- done_aphias[!duplicated(done_aphias)]
done_aphias <- done_aphias[!is.na(done_aphias)]
```

Get full file path:

```
files <- paste0("./t_matched_obis_recs/", files)
```

Get the indices of the files that we need for each dataset (Comte-Olden and Globtherm):

```
id_files_to_get_co <- which(done_aphias %in% unique(co_exp_dat$valid_AphiaID))
id_files_to_get_globtherm <- which(done_aphias %in% unique(globtherm_dat$valid_Aph
iaID))
```

This gives the following lists of files:

```
files_co <- files[id_files_to_get_co]
files_globtherm <- files[id_files_to_get_globtherm]
files_both <- files[which(
  done_aphias %in% unique(c(
    globtherm_dat$valid_AphiaID, co_exp_dat$valid_AphiaID)
    ))]
```

Set the variable types to read in, to ensure no parsing errors:

```
variable_type <- cols(
  "valid_AphiaID" = col_integer(),
  "decimalLongitude" = col_double(),
  "decimalLatitude" = col_double(),
  "depth" = col_double(),
  "eventDate" = col_datetime(format = ""),
  "scientificName" = col_character(),
  "aphiaID" = col_integer(),
  "year" = col_integer(),
  "month" = col_character(),
  "depth0" = col_double(),
  "grid_depth" = col_integer(),
  "grid_lon" = col_integer(),
  "grid_lat" = col_integer(),
  "iap_t" = col_double(),
  "iap_sst" = col_double(),
  "iap_sbt" = col_double(),
  "iap_grid_bottom_depth" = col_integer(),
  "bo_sst" = col_double(),
  "bo_sbt" = col_double()
  )
```

Read in all the files for each species as a single tibble for each dataset, and for both combined (might take a minute or two to read in all the files).

```
spp_occs_co <- files_co %>%
  map_df(~read_csv(., col_types = variable_type)) %>%
  distinct()

spp_occs_globtherm <- files_globtherm %>%
  map_df(~read_csv(., col_types = variable_type)) %>%
  distinct()

spp_occs_both <- files_both %>%
  map_df(~read_csv(., col_types = variable_type)) %>%
  distinct()
```

From these datasets we can get basic summary info, for instance the total number of OBIS records, and the range in year, depth, lon and lat of these records:

```
spp_occs_both %>%
  summarise(n_species = n_distinct(aphiaID),
            n_records = n(),
            first_year = min(year, na.rm = TRUE),
            last_year = max(year, na.rm = TRUE),
            min_depth = min(depth0, na.rm = TRUE),
            max_depth = max(depth0, na.rm = TRUE),
            min_lon = min(decimalLongitude, na.rm = TRUE),
            max_lon = max(decimalLongitude, na.rm = TRUE),
            min_lat = min(decimalLatitude, na.rm = TRUE),
            max_lat = max(decimalLatitude, na.rm = TRUE)
            )
```

```
## # A tibble: 1 x 10
##   n_species n_records first_year last_year min_depth max_depth min_lon
##       <int>     <int>      <int>     <int>     <dbl>     <dbl>   <dbl>
## 1       555   2176906       1643      2017         0      5870    -180
## # … with 3 more variables: max_lon <dbl>, min_lat <dbl>, max_lat <dbl>
```

We can also get the number and proportion of OBIS records not matched to temperature for each temperature dataset, as well numbers of records with no value for year or depth, using the `missing_var_summary` from `naniar`:

```
spp_occs_both %>% dplyr::select(year, depth, bo_sst, bo_sbt, iap_sst, iap_sbt, iap
_t) %>%
  miss_var_summary() %>%
  arrange(variable)
```

```
## # A tibble: 7 x 3
##   variable  n_miss pct_miss
##   <chr>      <int>    <dbl>
## 1 bo_sbt     80673     3.71
## 2 bo_sst     80297     3.69
## 3 depth    1021519    46.9
## 4 iap_sbt   404729    18.6
## 5 iap_sst   404729    18.6
## 6 iap_t     591076    27.2
## 7 year       95320     4.38
```

For the IAP matching it’s also important to know how many years (and what percentage) were outside the time scope of this dataset:

```
sum(spp_occs_both$year < 1941, na.rm = TRUE)
```

```
## [1] 17596
```

```
100*mean(spp_occs_both$year < 1941, na.rm = TRUE)
```

```
## [1] 0.845317
```

To look at similar stats for CO and Globtherm data separately:

```
spp_occs_co %>% dplyr::select(year, depth, bo_sst, bo_sbt, iap_sst, iap_sbt, iap_t
) %>%
  miss_var_summary() %>%
  arrange(variable)
```

```
## # A tibble: 7 x 3
##   variable n_miss pct_miss
##   <chr>     <int>    <dbl>
## 1 bo_sbt    19538     3.94
## 2 bo_sst    19542     3.94
## 3 depth    135873    27.4
## 4 iap_sbt  130128    26.2
## 5 iap_sst  130128    26.2
## 6 iap_t    156872    31.6
## 7 year      37714     7.61
```

```
spp_occs_globtherm %>% dplyr::select(year, depth, bo_sst, bo_sbt, iap_sst, iap_sbt
, iap_t) %>%
  miss_var_summary() %>%
  arrange(variable)
```

```
## # A tibble: 7 x 3
##   variable n_miss pct_miss
##   <chr>     <int>    <dbl>
## 1 bo_sbt    67564     3.71
## 2 bo_sst    67185     3.69
## 3 depth    909758    50.0
## 4 iap_sbt  297652    16.3
## 5 iap_sst  297652    16.3
## 6 iap_t    458722    25.2
## 7 year      60258     3.31
```

Map Occurrences

This adds all the occurrences to a 1 degree grid and ready to map them:

```
sp_oc_grid <- spp_occs_both %>%
  group_by(grid_lon, grid_lat) %>%
  summarise(n_recs = n()) %>%
    mutate(lat = grid_lat - 90, lon = ifelse(grid_lon > 180, grid_lon - 360, grid_
lon))
```

Alternatively you can read in this gridded dataset directly:

```
sp_oc_grid <- read_csv("data/all_spp_occs_grid1deg.csv")
```

The map uses a minimal black background theme:

```
theme_black_map = function(base_size = 12, base_family = "") {

  theme_grey(base_size = base_size, base_family = base_family) %+replace%

    theme(
      # Specify axis options
      axis.line = element_blank(),
      axis.text.x = element_blank(),
      axis.text.y = element_blank(),
      axis.ticks = element_blank(),
      axis.title.x = element_blank(),
      axis.title.y = element_blank(),
      # Specify legend options
      legend.background = element_rect(color = NA, fill = "black"),
      legend.key = element_rect(color = "black",  fill = "black"),
      legend.key.size = unit(1.2, "lines"),
      legend.key.height = NULL,
      legend.key.width = NULL,
      legend.text = element_text(size = base_size*0.8, color = "white"),
      legend.title = element_text(size = base_size*0.8, face = "bold", hjust = 0,
color = "white"),
      legend.position = "right",
      legend.text.align = NULL,
      legend.title.align = NULL,
      legend.direction = "vertical",
      legend.box = NULL,
      # Specify panel options
      panel.background = element_rect(fill = "black", color  =  NA),
      panel.border = element_rect(fill = NA, color = "grey50"),
      panel.grid.major = element_line(color = "grey35"),
      panel.grid.minor = element_line(color = "grey20"),
      panel.spacing = unit(0.5, "lines"),
      # Specify facetting options
      strip.background = element_rect(fill = "grey30", color = "grey10"),
      strip.text.x = element_text(size = base_size*0.8, color = "white"),
      strip.text.y = element_text(size = base_size*0.8, color = "white",angle = -9
0),
      # Specify plot options
      plot.background = element_rect(color = "black", fill = "black"),
      plot.title = element_text(size = base_size*1.2, color = "white"),
      plot.margin = unit(rep(1, 4), "lines")
     )
  }
```

Now produce the map using a coastlines map from the `maps` package, and save to file:

```
world_dat <- map_data("world")
obis_rec_summary_plot <- ggplot(sp_oc_grid, aes(x = lon, y = lat)) +
    geom_raster(aes(fill = log10(n_recs))) +
  scale_fill_viridis(name = expression(paste(log[10], N))) +
    geom_polygon(data = world_dat,
                 aes(x = long, y = lat, group = group), fill = "grey25") +
    theme_black_map() +
  theme(plot.caption = element_text(hjust = 0, size = 12))

ggsave("manuscript/figures/figure 1.png", plot = obis_rec_summary_plot,
       width = 9, height = 5, units = "in")
```

This is figure 1 in the ms, and shows the global distribution of the 2176906 occurrence records obtained from OBIS for 533 marine species with experimentally-derived thermal maxima available from our two data sources, mapped on a 1° grid.

Correlations between temperature affinity measures

```
co_t_corrs <- co_exp_dat %>% dplyr::select(
    bo_sst_mean, bo_sst_q95, bo_sbt_mean, bo_sbt_q95, bo_t_opt, bo_t_opt95,
    iap_t_mean, iap_t_q95, iap_sst_mean, iap_sst_q95, iap_sbt_mean, iap_sbt_q95) %
>%
  rename(bo_t_mean = bo_t_opt, bo_t_q95 = bo_t_opt95) %>%
    cor(use = "pairwise.complete") %>% round(digits = 2)
co_t_corrplot <-
  ggcorrplot(co_t_corrs, type = "lower", lab = TRUE, lab_size = 2) +
  theme(legend.position = "none")

globtherm_t_corrs <- globtherm_dat %>% dplyr::select(
    bo_sst_mean, bo_sst_q95, bo_sbt_mean, bo_sbt_q95, bo_t_opt, bo_t_opt95,
    iap_t_mean, iap_t_q95, iap_sst_mean, iap_sst_q95, iap_sbt_mean, iap_sbt_q95) %
>%
    rename(bo_t_mean = bo_t_opt, bo_t_q95 = bo_t_opt95) %>%
    cor(use = "pairwise.complete") %>% round(digits = 2)
globtherm_t_corrplot <-
  ggcorrplot(globtherm_t_corrs, type = "lower", lab = TRUE, lab_size = 2) +
  theme(legend.position = "none")

plot_grid(co_t_corrplot, globtherm_t_corrplot, nrow = 1, ncol = 2, labels = "auto"
)
```

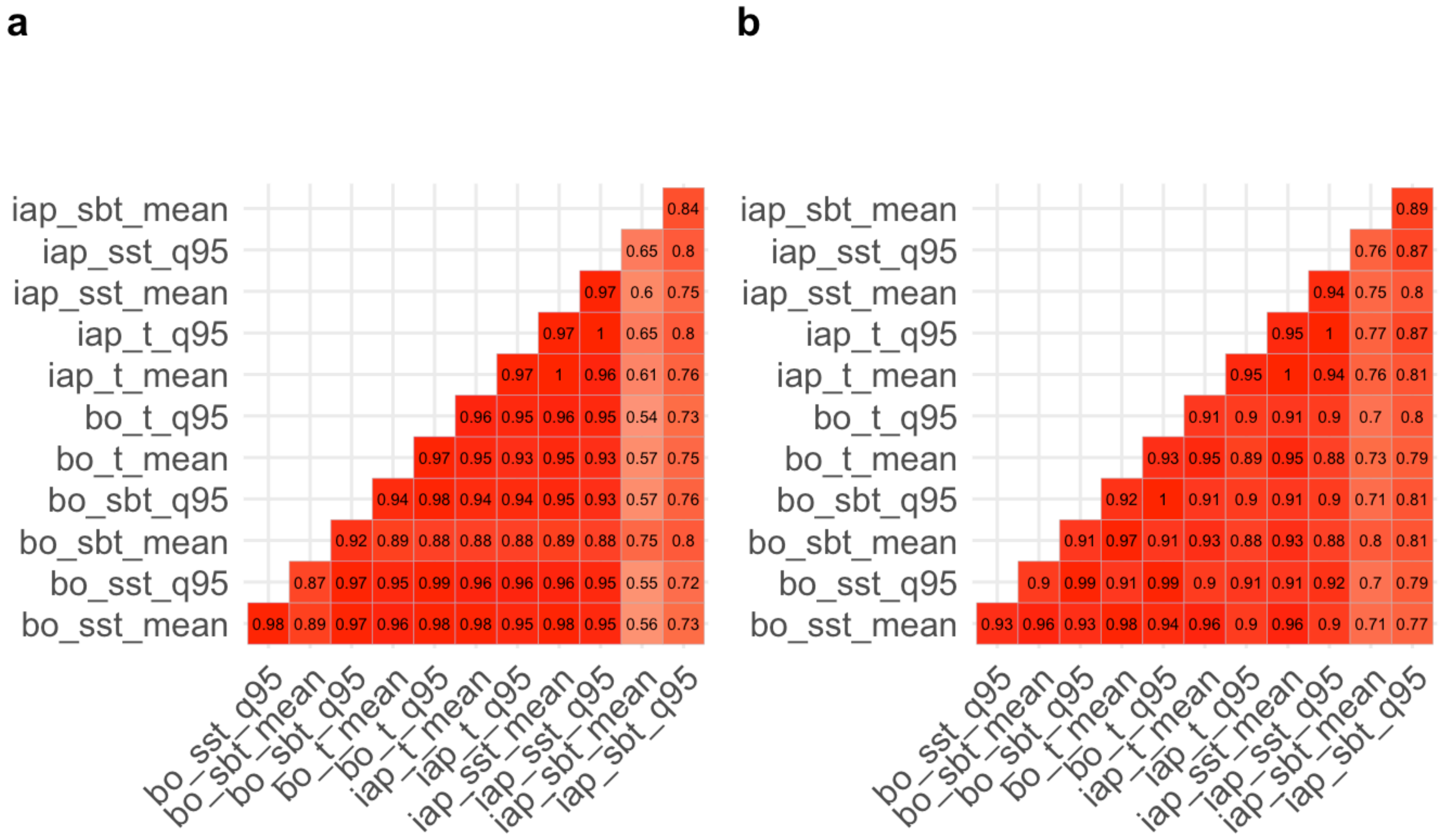

Figure S2 Correlations between different estimates of temperature affinity for species in the Comte-Olden dataset (*a*) and in the Globtherm dataset (*b*). In each case, 'bo' indicates temperature affinity derived from bio-ORACLE gridded temerature data, IAP is the IAP gridded temperature data, 'sbt' is Sea Bottom Tempeature, 'sst' is Sea Surface Temperature, and 't' is temperature at depth of sample (for IAP), or the 'optimum' temperature - SBT or SST depending of species habitat affinity (for BO). Temperature affinity summaries shown here are means ('mean') and upper 95th percentile ('q95'). Other summary statistics (e.g. median) show similar patterns.

Relationships between experimentally-derived thermal limits and occurrence-derived thermal affinities

This code generates the four panels of Fig 2 in the ms. Initial data exploration revealed two significant outliers in the Globtherm dataset, with temperature affinities <10C but critical upper temperatuers >40C:

```
globtherm_dat %>%
  mutate(delta_t = T_max - bo_t_opt) %>%
  filter(delta_t > 40) %>%
  dplyr::select(
    valid_AphiaID, sciname, T_max, bo_t_opt, iap_t_mean, REF_max, fun_grp, records
)
```

```
## # A tibble: 2 x 8
##   valid_AphiaID sciname  T_max bo_t_opt iap_t_mean REF_max  fun_grp records
##           <dbl> <chr>    <dbl>    <dbl>      <dbl> <chr>    <chr>     <dbl>
## 1        508323 Halozet…  40.1    -1.08      -1.71 Deere_e… benthos       2
## 2        145770 Devaler…  50       6.41      11.4  Bischof… macroa…     148
```

Checking back to the original references given in GlobTherms for these two species, the T_max value for *Halozetes belgicae* is as reported in Deere et al. 2006 (http://dx.doi.org/10.1016/j.jinsphys.2006.03.009 (http://dx.doi.org/10.1016/j.jinsphys.2006.03.009)), however these authors report this species as occurring in the supra-littoral zone, so although it is reported as 'marine' by WoRMS (see http://www.marinespecies.org/aphia.php?p=taxdetails&id=508323 (http://www.marinespecies.org/aphia.php?p=taxdetails&id=508323)), air temperature is probably more appropriate than sea temperature for this species. However, we retain the species in our dataset here. Checking Bischoff & Wiencke (1993) for *Devaleraea ramentacea* gives a maximum survival temperature for this species of 19C, so we update Tmax to 19 for this species in globtherm_dat:

```
globtherm_dat$T_max[globtherm_dat$valid_AphiaID == 145770] <- 19
```

Now produce the figure. First, T-max v 'optimal' (SST or SBT depending on species habitat groiup) Bio-ORACLE thermal affinity for Comte-Olden species:

```
bo_t_opt_mean_v_ct_exp_co <- ggplot(co_exp_dat, aes(y = CT_max, x = bo_t_opt)) +
   geom_smooth(aes(group = habitat, colour = habitat),
               se = FALSE, method = lm, formula = y ~ poly(x, 2), size = 0.5) +
   geom_errorbar(aes(ymax = max_CT_max, ymin = min_CT_max, width=0, colour = habit
at),
                 alpha = 0.2) +
   geom_errorbarh(aes(
     xmax = bo_t_opt + bo_t_opt_sd, xmin = bo_t_opt - bo_t_opt_sd, colour = habita
t),
     alpha = 0.2) +
   geom_point(aes(fill = habitat), pch = 21, alpha = 0.5) +
   scale_colour_discrete(
     breaks = c("demersal", "benthopelagic", "reef-associated", "pelagic")) +
   scale_fill_discrete(
     breaks = c("demersal", "benthopelagic", "reef-associated", "pelagic")) +
   geom_smooth(colour = "black", se = FALSE, method = lm, formula = y ~ poly(x, 2)
) +
   geom_abline(slope = 1, intercept = 0, linetype = "dashed") +
   theme_bw() +
   xlab("Mean Bio-ORACLE 'best' T") +
   ylab(expression(T[max])) +
   ylim(7, 45) +
   ggtitle("A") + theme(plot.title = element_text(hjust = 0)) +
   theme(legend.position = c(0.85, 0.2))
```

Second, IAP temperature at depth for Comte-Olden species:

```
iap_t_mean_v_ct_exp_co <- ggplot(co_exp_dat, aes(y = CT_max, x = iap_t_mean)) +
  geom_smooth(aes(group = habitat, colour = habitat),
              se = FALSE, method = lm, formula = y ~ poly(x, 2), size = 0.5) +
  geom_errorbar(aes(
    ymax = max_CT_max, ymin = min_CT_max, width=0, colour = habitat),
                alpha = 0.2) +
  geom_errorbarh(aes(
    xmax = iap_t_mean + iap_t_sd, xmin = iap_t_mean - iap_t_sd, colour = habitat),
        alpha = 0.2) +
  geom_point(aes(fill = habitat), pch = 21, alpha = 0.5) +
  scale_colour_discrete(
    breaks = c("demersal", "benthopelagic", "reef-associated", "pelagic")) +
  scale_fill_discrete(
    breaks = c("demersal", "benthopelagic", "reef-associated", "pelagic")) +
  geom_smooth(colour = "black", se = FALSE, method = lm, formula = y ~ poly(x, 2))
+
  geom_abline(slope = 1, intercept = 0, linetype = "dashed") +
    theme_bw() +
    xlab("Mean IAP T-at-depth") +
    ylab(expression(T[max])) +
    ylim(7, 45) +
    ggtitle("B") + theme(plot.title = element_text(hjust = 0)) +
    theme(legend.position = "none")
```

Next, optimum BioORACLE temperature for GlobTherm species:

```
bo_t_opt_mean_v_t_exp_glob <- ggplot(globtherm_dat, aes(y = T_max, x = bo_t_opt))
+
  geom_smooth(
    data = filter(globtherm_dat, !(fun_grp %in% c("birds", "mammals", "nekton"))),
              aes(group = fun_grp, colour = fun_grp),
              se = FALSE, method = lm, formula = y ~ poly(x, 2), size = 0.5) +
  geom_errorbarh(data = globtherm_dat, aes(
    xmax = bo_t_opt + bo_t_opt_sd, xmin = bo_t_opt - bo_t_opt_sd,
    colour = fun_grp),
        alpha = 0.2) +
    geom_point(aes(fill = fun_grp), pch = 21, alpha = 0.5) +
    scale_colour_discrete(name = "functional group") +
    scale_fill_discrete(name = "functional group") +
    geom_smooth(colour = "black", se = FALSE, method = lm, formula = y ~ poly(x, 2
)) +
    geom_abline(slope = 1, intercept = 0, linetype = "dashed") +
    theme_bw() +
    xlab("Mean Bio-ORACLE 'best' T") +
    ylab(expression(T[max])) +
    ylim(0, 45) +
    xlim(-2, 30) +
    ggtitle("C") + theme(plot.title = element_text(hjust = 0)) +
    theme(legend.position = c(0.85, 0.2))
```

Finaly, mean IAP T-at-depth for Globtherm species:

```
iap_t_mean_v_t_exp_glob <- ggplot(globtherm_dat, aes(y = T_max, x = iap_t_mean)) +
    geom_smooth(
      data = filter(globtherm_dat, !(fun_grp %in% c("birds", "mammals", "nekton"))
),
               aes(group = fun_grp, colour = fun_grp),
               se = FALSE, method = lm, formula = y ~ poly(x, 2), size = 0.5) +
    geom_errorbarh(aes(xmax = iap_t_mean + iap_t_sd, xmin = iap_t_mean - iap_t_sd,
                       colour = fun_grp),
                   alpha = 0.2) +
    geom_point(aes(fill = fun_grp), pch = 21, alpha = 0.5) +
    geom_smooth(colour = "black", se = FALSE, method = lm, formula = y ~ poly(x, 2
)) +
    geom_abline(slope = 1, intercept = 0, linetype = "dashed") +
    theme_bw() +
    xlab("Mean IAP T-at-depth") +
    ylab(expression(T[max])) +
    ylim(0, 45) +
    xlim(-2, 30) +
    ggtitle("D") + theme(plot.title = element_text(hjust = 0)) +
    theme(legend.position = "none")
```

To assemble Figure 2:

```
comb_fig2 <- plot_grid(
  bo_t_opt_mean_v_ct_exp_co,
  iap_t_mean_v_ct_exp_co,
  bo_t_opt_mean_v_t_exp_glob,
  iap_t_mean_v_t_exp_glob,
  nrow = 2, ncol = 2)
ggsave("manuscript/figures/figure 2.png", comb_fig2,
       width = 12, height = 12, units = "in")
```

This is Figure 2 in the ms, and shows experimentally-derived critical thermal limits (Tmax) for 157 (A), and 154 (B) marine fish species taken from Comte & Olden, and for 420 (C) and 403 (D) marine species taken from the GlobTherm, against thermal affinity calculated from a combination of Bio-ORACLE Sea Surface Temperature (SST) and Sea Bottom Temperature (SBT) depending on fish habitat or functional group, at c. 9km resolution (A, C), and date (month-year)-matched IAP gridded temperature at sample depth at 1 degree resolution (B, D). Points are means, error bars are min and max reported values for Tmax (where available) and standard deviations for temperature affinities. In each case, separate 2nd order polynomials are shown for each of the habitat associations (A, B) or functional groups (C, D - NB we do not fit separate models for birds (n = 9), mammals (n = 5), or nekton (n = 5), due to small sample sizes) (coloured lines), together with a single 2nd order polynomial fitted across all species (solid black line). The 1:1 relationship is shown as a dashed line.

To fit the statistical models associated with these figures, first for C-O data and BO temperature:

```
ct_v_bo_t_co <- lm(CT_max ~ bo_t_opt + I(bo_t_opt^2),
                   data = subset(co_exp_dat, !is.na(bo_t_opt)))
summary(ct_v_bo_t_co)
```

```
##
## Call:
## lm(formula = CT_max ~ bo_t_opt + I(bo_t_opt^2), data = subset(co_exp_dat,
##     !is.na(bo_t_opt)))
##
## Residuals:
##     Min      1Q  Median      3Q     Max
## -7.5713 -1.9057 -0.0662  2.0009 11.5484
##
## Coefficients:
##                Estimate Std. Error t value Pr(>|t|)
## (Intercept)   15.681917   0.765753  20.479  < 2e-16 ***
## bo_t_opt       1.502415   0.106428  14.117  < 2e-16 ***
## I(bo_t_opt^2) -0.024922   0.003331  -7.483  5.2e-12 ***
## ---
## Signif. codes:  0 '***' 0.001 '**' 0.01 '*' 0.05 '.' 0.1 ' ' 1
##
## Residual standard error: 3.062 on 154 degrees of freedom
## Multiple R-squared:  0.8294, Adjusted R-squared:  0.8272
## F-statistic: 374.3 on 2 and 154 DF,  p-value: < 2.2e-16
```

Add habitat and its interaction with T:

```
ct_v_bo_t_co_hab <- lm(CT_max ~ poly(bo_t_opt, 2) * habitat,
    data = subset(co_exp_dat, !is.na(bo_t_opt)))
summary.aov(ct_v_bo_t_co_hab)
```

```
##                              Df Sum Sq Mean Sq F value Pr(>F)
## poly(bo_t_opt, 2)             2   7017    3508 392.779 <2e-16 ***
## habitat                       3     83      28   3.084 0.0293 *
## poly(bo_t_opt, 2):habitat     6     66      11   1.226 0.2963
## Residuals                   145   1295       9
## ---
## Signif. codes:  0 '***' 0.001 '**' 0.01 '*' 0.05 '.' 0.1 ' ' 1
```

No evidence of an interaction between T and habitat, so try removing interaction:

```
ct_v_bo_t_co_hab_add <- lm(CT_max ~ bo_t_opt + I(bo_t_opt^2) + habitat,
    data = subset(co_exp_dat, !is.na(bo_t_opt)))
summary.aov(ct_v_bo_t_co_hab_add)
```

```
##                 Df Sum Sq Mean Sq F value   Pr(>F)
## bo_t_opt         1   6492    6492 720.327  < 2e-16 ***
## I(bo_t_opt^2)    1    525     525  58.239 2.44e-12 ***
## habitat          3     83      28   3.057   0.0302 *
## Residuals      151   1361       9
## ---
## Signif. codes:  0 '***' 0.001 '**' 0.01 '*' 0.05 '.' 0.1 ' ' 1
```

There is a significant main effect of habitat (P = 0.0302), we can get examine the coefficients and their 95% CIsfor each habitat group:

```
ci_mat <- rbind(confint(ct_v_bo_t_co_hab_add)[1,],
                coef(ct_v_bo_t_co_hab_add)[1] + confint(ct_v_bo_t_co_hab_add)[c(4:
6),])
ct_v_bo_t_co_hab_add_coefs <- tibble(
  habitat = sort(unique(co_exp_dat$habitat)),
  coefs = c(coef(ct_v_bo_t_co_hab_add)[1],
            coef(ct_v_bo_t_co_hab_add)[1] + coef(ct_v_bo_t_co_hab_add)[4:6]),
  lower_ci = ci_mat[, 1],
  upper_ci = ci_mat[, 2]
)
ct_v_bo_t_co_hab_add_coefs
```

```
## # A tibble: 4 x 4
##   habitat         coefs lower_ci upper_ci
##   <chr>           <dbl>    <dbl>    <dbl>
## 1 benthopelagic    16.2     14.2     18.2
## 2 demersal         15.9     14.2     17.5
## 3 pelagic          13.4     11.1     15.7
## 4 reef-associated  14.4     12.4     16.5
```

These CIs generally overlap indicating little evidence for a substantial habitat effect, see also the AIC scores from the three models:

```
AIC(ct_v_bo_t_co, ct_v_bo_t_co_hab, ct_v_bo_t_co_hab_add)
```

```
##                       df      AIC
## ct_v_bo_t_co           4 801.8684
## ct_v_bo_t_co_hab      13 802.8430
## ct_v_bo_t_co_hab_add   7 798.6122
```

Including habitat decreases AIC by 3.26 over the next best fitting model, but there is only a small change in R2, with the model including habitat having R2 = 0.84 cf. 0.83 for the model not including habitat, and 0.85 for the model allowing habitat and T to interact. Across all habitats, the relationship between T and T_max is hump-shaped:

```
t_coefs <- tibble(
  var = c("T", "T^2"),
  coef = coef(ct_v_bo_t_co_hab_add)[2:3],
  lower_ci = confint(ct_v_bo_t_co_hab_add)[2:3, 1],
  upper_ci = confint(ct_v_bo_t_co_hab_add)[2:3, 2]
)
t_coefs
```

```
## # A tibble: 2 x 4
##   var      coef lower_ci upper_ci
##   <chr>   <dbl>    <dbl>    <dbl>
## 1 T      1.46     1.24     1.68
## 2 T^2   -0.0219  -0.0294  -0.0144
```

The maximum of this function occurs at a temperature affinity of 33.38degC.

Now do the same for C-O data and IAP temperature:

```
ct_v_iap_t_co <- lm(CT_max ~ iap_t_mean + I(iap_t_mean^2),
                   data = subset(co_exp_dat, !is.na(iap_t_mean)))
summary(ct_v_iap_t_co)
```

```
##
## Call:
## lm(formula = CT_max ~ iap_t_mean + I(iap_t_mean^2), data = subset(co_exp_dat,
##     !is.na(iap_t_mean)))
##
## Residuals:
##    Min     1Q Median     3Q    Max
## -7.301 -1.753 -0.329  1.855  8.411
##
## Coefficients:
##                 Estimate Std. Error t value Pr(>|t|)
## (Intercept)     14.54768    0.73927  19.679  < 2e-16 ***
## iap_t_mean       1.37848    0.09883  13.948  < 2e-16 ***
## I(iap_t_mean^2) -0.01870    0.00302  -6.193 5.34e-09 ***
## ---
## Signif. codes:  0 '***' 0.001 '**' 0.01 '*' 0.05 '.' 0.1 ' ' 1
##
## Residual standard error: 2.749 on 151 degrees of freedom
## Multiple R-squared:  0.8648, Adjusted R-squared:  0.863
## F-statistic: 482.7 on 2 and 151 DF,  p-value: < 2.2e-16
```

Add habitat and its interaction with T:

```
ct_v_iap_t_co_hab <- lm(CT_max ~ poly(iap_t_mean, 2) * habitat,
    data = subset(co_exp_dat, !is.na(iap_t_mean)))
summary.aov(ct_v_iap_t_co_hab)
```

```
##                              Df Sum Sq Mean Sq F value Pr(>F)
## poly(iap_t_mean, 2)           2   7296    3648 478.773 <2e-16 ***
## habitat                       3      6       2   0.252  0.860
## poly(iap_t_mean, 2):habitat   6     53       9   1.167  0.328
## Residuals                   142   1082       8
## ---
## Signif. codes:  0 '***' 0.001 '**' 0.01 '*' 0.05 '.' 0.1 ' ' 1
```

No evidence of an interaction between T and habitat, so try removing interaction:

```
ct_v_iap_t_co_hab_add <- lm(CT_max ~ iap_t_mean + I(iap_t_mean^2) + habitat,
    data = subset(co_exp_dat, !is.na(iap_t_mean)))
summary.aov(ct_v_iap_t_co_hab_add)
```

```
##                  Df Sum Sq Mean Sq F value Pr(>F)
## iap_t_mean        1   7006    7006 913.348 <2e-16 ***
## I(iap_t_mean^2)   1    290     290  37.777  7e-09 ***
## habitat           3      6       2   0.251  0.861
## Residuals       148   1135       8
## ---
## Signif. codes:  0 '***' 0.001 '**' 0.01 '*' 0.05 '.' 0.1 ' ' 1
```

There is no significant main effect of habitat (P = 0.8607), the model not including habitat has an R2 value (0.86) only marginally smaller than the more complicated models (for the additive model, R2 = 0.87, and for the interactive model, R2 = 0.87); finally, the model that does not include habitat has the lowest AIC:

```
AIC(ct_v_iap_t_co, ct_v_iap_t_co_hab, ct_v_iap_t_co_hab_add)
```

```
##                       df      AIC
## ct_v_iap_t_co          4 753.4583
## ct_v_iap_t_co_hab     13 763.2680
## ct_v_iap_t_co_hab_add  7 758.6777
```

So we can treat all species together, and find the overall relationship between T and T_max. Again, across all habitats the relationship between T and T_max is hump-shaped:

```
t_coefs <- tibble(
  var = c("T", "T^2"),
  coef = coef(ct_v_iap_t_co)[2:3],
  lower_ci = confint(ct_v_iap_t_co)[2:3, 1],
  upper_ci = confint(ct_v_iap_t_co)[2:3, 2]
)
t_coefs
```

```
## # A tibble: 2 x 4
##   var      coef lower_ci upper_ci
##   <chr>   <dbl>    <dbl>    <dbl>
## 1 T      1.38     1.18     1.57
## 2 T^2   -0.0187  -0.0247  -0.0127
```

The maximum of this function occurs at a temperature affinity of 36.86degC.

Now do the same for Globtherm data and BO temperature:

```
ct_v_bo_t_glob <- lm(T_max ~ bo_t_opt + I(bo_t_opt^2),
                     data = subset(globtherm_dat, !is.na(bo_t_opt)))
summary(ct_v_bo_t_glob)
```

```
##
## Call:
## lm(formula = T_max ~ bo_t_opt + I(bo_t_opt^2), data = subset(globtherm_dat,
##     !is.na(bo_t_opt)))
##
## Residuals:
##      Min       1Q   Median       3Q      Max
## -12.5186  -3.1785   0.0601   3.1002  26.3670
##
## Coefficients:
##                Estimate Std. Error t value Pr(>|t|)
## (Intercept)   15.277662   0.742850  20.566  < 2e-16 ***
## bo_t_opt       1.411061   0.108467  13.009  < 2e-16 ***
## I(bo_t_opt^2) -0.020723   0.003602  -5.753 1.76e-08 ***
## ---
## Signif. codes:  0 '***' 0.001 '**' 0.01 '*' 0.05 '.' 0.1 ' ' 1
##
## Residual standard error: 4.774 on 397 degrees of freedom
##   (20 observations deleted due to missingness)
## Multiple R-squared:  0.6168, Adjusted R-squared:  0.6149
## F-statistic: 319.5 on 2 and 397 DF,  p-value: < 2.2e-16
```

Add functional group and its interaction with T:

```
ct_v_bo_t_glob_fg <- lm(T_max ~ poly(bo_t_opt, 2, raw = TRUE) * fun_grp,
    data = subset(globtherm_dat, !is.na(bo_t_opt)))
summary.aov(ct_v_bo_t_glob_fg)
```

```
##                                        Df Sum Sq Mean Sq F value  Pr(>F)
## poly(bo_t_opt, 2, raw = TRUE)           2  14564    7282 337.083 < 2e-16
## fun_grp                                 5    373      75   3.456 0.00456
## poly(bo_t_opt, 2, raw = TRUE):fun_grp   8    379      47   2.195 0.02712
## Residuals                             384   8296      22
##
## poly(bo_t_opt, 2, raw = TRUE)         ***
## fun_grp                               **
## poly(bo_t_opt, 2, raw = TRUE):fun_grp *
## Residuals
## ---
## Signif. codes:  0 '***' 0.001 '**' 0.01 '*' 0.05 '.' 0.1 ' ' 1
## 20 observations deleted due to missingness
```

There is evidence of an interaction between T and habitat (P = 0.0271, but for completeness fit a model without the interaction:

```
ct_v_bo_t_glob_fg_add <- lm(T_max ~ bo_t_opt + I(bo_t_opt^2) + fun_grp,
    data = subset(globtherm_dat, !is.na(bo_t_opt)))
summary.aov(ct_v_bo_t_glob_fg_add)
```

```
##                 Df Sum Sq Mean Sq F value   Pr(>F)    
## bo_t_opt         1  13810   13810 624.039  < 2e-16 ***
## I(bo_t_opt^2)    1    754     754  34.081 1.11e-08 ***
## fun_grp          5    373      75   3.374  0.00537 ** 
## Residuals      392   8675      22                     
## ---
## Signif. codes:  0 '***' 0.001 '**' 0.01 '*' 0.05 '.' 0.1 ' ' 1
## 20 observations deleted due to missingness
```

This indicates a significant main effect of habitat (P = 0.0054). Comparing AIC scores from the three models:

```
(glob_bo_aics <- AIC(ct_v_bo_t_glob, ct_v_bo_t_glob_fg, ct_v_bo_t_glob_fg_add) %>%
  rownames_to_column(var = "model") %>%
  arrange(AIC)
)
```

```
##                   model df      AIC
## 1     ct_v_bo_t_glob_fg 17 2381.965
## 2 ct_v_bo_t_glob_fg_add  9 2383.848
## 3        ct_v_bo_t_glob  4 2390.703
```

The model including the interaction has an AIC 1.883 smaller than the next best model, indicating a marignal improvement in fit, which we can also see in the R2 values (interactive model: 0.65, additive model: 0.63, model without functional group: 0.62). To interpret the coefficients of the model with interaction:

```
ci_mat_glob <- rbind(confint(ct_v_bo_t_glob_fg)[1,],
                coef(ct_v_bo_t_glob_fg)[1] + confint(ct_v_bo_t_glob_fg)[c(4:8),],
                confint(ct_v_bo_t_glob_fg)[2,],
                coef(ct_v_bo_t_glob_fg)[2] +
                  confint(ct_v_bo_t_glob_fg)[c(9,11,13,15,17),],
                confint(ct_v_bo_t_glob_fg)[3,],
                coef(ct_v_bo_t_glob_fg)[3] +
                  confint(ct_v_bo_t_glob_fg)[c(10,12,14,16,18),]
                )
(ct_v_bo_t_glob_fg_coefs <- tibble(
  fun_grp = rep(sort(unique(globtherm_dat$fun_grp)), 3),
  coef_type = rep(c("intercept", "T", "T^2"), each = 6),
  coefs = c(coef(ct_v_bo_t_glob_fg)[1],
            coef(ct_v_bo_t_glob_fg)[1] + coef(ct_v_bo_t_glob_fg)[4:8],
            coef(ct_v_bo_t_glob_fg)[2],
            coef(ct_v_bo_t_glob_fg)[2] + coef(ct_v_bo_t_glob_fg)[c(9,11,13,15,17)]
,
            coef(ct_v_bo_t_glob_fg)[3],
            coef(ct_v_bo_t_glob_fg)[3] + coef(ct_v_bo_t_glob_fg)[c(10,12,14,16,18)
]),
  lower_ci = ci_mat_glob[, 1],
  upper_ci = ci_mat_glob[, 2]
  )
)
```

```
## # A tibble: 18 x 5
##    fun_grp    coef_type     coefs lower_ci  upper_ci
##    <chr>      <chr>         <dbl>    <dbl>     <dbl>
##  1 benthos    intercept  14.6      12.5     16.7
##  2 birds      intercept -32.2     -76.8     12.4
##  3 fish       intercept  14.1      10.1     18.0
##  4 macroalgae intercept  17.0      13.8     20.2
##  5 mammals    intercept   1.36     -7.89    10.6
##  6 nekton     intercept  55.1     -63.8    174.
##  7 benthos    T           1.66      1.31     2.02
##  8 birds      T          11.7       3.02    20.4
##  9 fish       T           1.84      1.25     2.43
## 10 macroalgae T           1.06      0.558    1.57
## 11 mammals    T          NA        NA       NA
## 12 nekton     T          -2.52    -16.1     11.1
## 13 benthos    T^2        -0.0291   -0.0428  -0.0153
## 14 birds      T^2        -0.488    -0.856   -0.120
## 15 fish       T^2        -0.0348   -0.0546  -0.0150
## 16 macroalgae T^2        -0.00971  -0.0282   0.00875
## 17 mammals    T^2        NA        NA       NA
## 18 nekton     T^2         0.0679   -0.268    0.404
```

Note the wide confidence intervals for coefficients associated with groups with small sample sizes (birds, mammals, nekton). But for the groups with a reasonable sample size, we observe a hump-shaped relationship, except in macroalgae where the CI of the T^2 coefficient includes 0 (-0.028 to 0.009).

Finally, for Globtherm data and IAP temperature:

```
ct_v_iap_t_glob <- lm(T_max ~ iap_t_mean + I(iap_t_mean^2),
                  data = subset(globtherm_dat, !is.na(iap_t_mean)))
summary(ct_v_iap_t_glob)
```

```
##
## Call:
## lm(formula = T_max ~ iap_t_mean + I(iap_t_mean^2), data = subset(globtherm_dat,
##     !is.na(iap_t_mean)))
##
## Residuals:
##      Min       1Q   Median       3Q      Max
## -12.2776  -3.2032  -0.0159   2.9456  27.9130
##
## Coefficients:
##                  Estimate Std. Error t value Pr(>|t|)
## (Intercept)     14.308006   0.808985  17.686  < 2e-16 ***
## iap_t_mean       1.221046   0.105138  11.614  < 2e-16 ***
## I(iap_t_mean^2) -0.012562   0.003358  -3.741 0.000211 ***
## ---
## Signif. codes:  0 '***' 0.001 '**' 0.01 '*' 0.05 '.' 0.1 ' ' 1
##
## Residual standard error: 4.786 on 380 degrees of freedom
##   (20 observations deleted due to missingness)
## Multiple R-squared:  0.6201, Adjusted R-squared:  0.6181
## F-statistic: 310.1 on 2 and 380 DF,  p-value: < 2.2e-16
```

Add functional group and its interaction with T:

```
ct_v_iap_t_glob_fg <- lm(T_max ~ poly(iap_t_mean, 2, raw = TRUE) * fun_grp,
    data = subset(globtherm_dat, !is.na(iap_t_mean)))
summary.aov(ct_v_iap_t_glob_fg)
```

```
##                                          Df Sum Sq Mean Sq F value
## poly(iap_t_mean, 2, raw = TRUE)           2  14210    7105 332.840
## fun_grp                                   5    486      97   4.552
## poly(iap_t_mean, 2, raw = TRUE):fun_grp   8    385      48   2.256
## Residuals                               367   7834      21
##                                           Pr(>F)
## poly(iap_t_mean, 2, raw = TRUE)          < 2e-16 ***
## fun_grp                                 0.000485 ***
## poly(iap_t_mean, 2, raw = TRUE):fun_grp 0.023075 *
## Residuals
## ---
## Signif. codes:  0 '***' 0.001 '**' 0.01 '*' 0.05 '.' 0.1 ' ' 1
## 20 observations deleted due to missingness
```

Again there is evidence of an interaction between T and habitat (P = 0.0231, but for completeness fit a model without the interaction:

```
ct_v_iap_t_glob_fg_add <- lm(T_max ~ iap_t_mean + I(iap_t_mean^2) + fun_grp,
    data = subset(globtherm_dat, !is.na(iap_t_mean)))
summary.aov(ct_v_iap_t_glob_fg_add)
```

```
##                 Df Sum Sq Mean Sq F value    Pr(>F)
## iap_t_mean       1  13890   13890 633.677  < 2e-16 ***
## I(iap_t_mean^2)  1    321     321  14.629 0.000153 ***
## fun_grp          5    486      97   4.434 0.000617 ***
## Residuals      375   8220      22
## ---
## Signif. codes:  0 '***' 0.001 '**' 0.01 '*' 0.05 '.' 0.1 ' ' 1
## 20 observations deleted due to missingness
```

This indicates a significant main effect of habitat (P = 610^{-4}). Comparing AIC scores from the three models:

```
(glob_iap_aics <- AIC(ct_v_iap_t_glob, ct_v_iap_t_glob_fg, ct_v_iap_t_glob_fg_add)
%>%
  rownames_to_column(var = "model") %>%
  arrange(AIC)
)
```

```
##                    model df      AIC
## 1     ct_v_iap_t_glob_fg 17 2276.894
## 2 ct_v_iap_t_glob_fg_add  9 2279.282
## 3        ct_v_iap_t_glob  4 2291.279
```

The model including the interaction has an AIC 2.388 smaller than the next best model, indicating a marignal improvement in fit, which we can also see in the R2 values (interactive model: 0.66, additive model: 0.64, model without functional group: 0.62). To interpret the coefficients of the model with interaction:

```
ci_mat_glob <- rbind(confint(ct_v_iap_t_glob_fg)[1,],
                coef(ct_v_iap_t_glob_fg)[1] + confint(ct_v_iap_t_glob_fg)[c(4:8),]
,
                confint(ct_v_iap_t_glob_fg)[2,],
                coef(ct_v_iap_t_glob_fg)[2] +
                  confint(ct_v_iap_t_glob_fg)[c(9,11,13,15,17),],
                confint(ct_v_iap_t_glob_fg)[3,],
                coef(ct_v_iap_t_glob_fg)[3] +
                  confint(ct_v_iap_t_glob_fg)[c(10,12,14,16,18),]
                )

(ct_v_iap_t_glob_fg_coefs <- tibble(
  fun_grp = rep(sort(unique(globtherm_dat$fun_grp)), 3),
  coef_type = rep(c("intercept", "T", "T^2"), each = 6),
  coefs = c(coef(ct_v_iap_t_glob_fg)[1],
            coef(ct_v_iap_t_glob_fg)[1] + coef(ct_v_iap_t_glob_fg)[4:8],
            coef(ct_v_iap_t_glob_fg)[2],
            coef(ct_v_iap_t_glob_fg)[2] + coef(ct_v_iap_t_glob_fg)[c(9,11,13,15,17
)],
            coef(ct_v_iap_t_glob_fg)[3],
            coef(ct_v_iap_t_glob_fg)[3] + coef(ct_v_iap_t_glob_fg)[c(10,12,14,16,1
8)]),
  lower_ci = ci_mat_glob[, 1],
  upper_ci = ci_mat_glob[, 2]
  )
)
```

```
## # A tibble: 18 x 5
##    fun_grp    coef_type    coefs lower_ci  upper_ci
##    <chr>      <chr>        <dbl>    <dbl>     <dbl>
##  1 benthos    intercept  14.1      11.9     16.2
##  2 birds      intercept -15.1     -46.9     16.7
##  3 fish       intercept  12.4       8.19    16.6
##  4 macroalgae intercept  15.6      12.1     19.1
##  5 mammals    intercept   0.520    -8.69     9.73
##  6 nekton     intercept  33.0     -89.4    155.
##  7 benthos    T           1.48      1.14     1.82
##  8 birds      T           9.65      2.45    16.9
##  9 fish       T           1.73      1.16     2.30
## 10 macroalgae T           0.912     0.425    1.40
## 11 mammals    T          NA        NA       NA
## 12 nekton     T          -0.0295  -12.8     12.7
## 13 benthos    T^2        -0.0212   -0.0335  -0.00883
## 14 birds      T^2        -0.447    -0.794   -0.100
## 15 fish       T^2        -0.0287   -0.0471  -0.0103
## 16 macroalgae T^2        -0.00238  -0.0191   0.0144
## 17 mammals    T^2        NA        NA       NA
## 18 nekton     T^2         0.00655  -0.296    0.309
```

Note again the wide confidence intervals for coefficients associated with groups with small sample sizes (birds, mammals, nekton). But for the groups with a reasonable sample size, we observe a hump-shaped relationship, except in macroalgae where the CI of the T^2 coefficient includes 0 (-0.019 to 0.014).

Finally, to produce figure 3, combine data from globtherm and CO, adding an identifyer for each data source, and creating a 'habitat' variable in Globtherm to match the habitat variable in CO. Then simplify functional groups, and for clarity exclude the outlying benthic species (Halozetes belgicae, see discussion of outlying points above):

```
co_dat_temp <- dplyr::select(
  co_exp_dat, valid_AphiaID, CT_max, iap_t_mean, habitat) %>%
  mutate(ds_id = "CO")
globtherm_dat_temp <- globtherm_dat %>% mutate(
  habitat = case_when(!is.na(DemersPelag) ~ DemersPelag, TRUE ~ fun_grp)) %>%
  dplyr::select(valid_AphiaID, T_max, iap_t_mean, habitat) %>%
  mutate(ds_id = "Glob") %>%
  rename(CT_max = T_max)

cross_source_dat <- bind_rows(co_dat_temp, globtherm_dat_temp) %>%
  distinct(valid_AphiaID, .keep_all = TRUE) %>%
  filter(!(habitat %in% c("birds", "mammals", "nekton"))) %>%
  mutate(delta_t = CT_max - iap_t_mean,
         fgroup = case_when(
           habitat %in%
             c("pelagic-neritic", "pelagic-oceanic", "pelagic") ~ "pelagic fish",
           habitat %in% "demersal" ~ "demersal fish",
           habitat %in% "benthopelagic" ~ "benthopelagic fish",
           habitat == "reef-associated" ~ "reef fish",
           TRUE ~ habitat
         ),
         fgroup2 = case_when(
           fgroup %in%
             c("pelagic fish", "demersal fish",
               "reef fish", "benthopelagic fish") ~ "fish",
           TRUE ~ fgroup
         ),
         fgroup = factor(fgroup,
                         levels = c(
                           "benthos", "demersal fish",
                           "benthopelagic fish", "pelagic fish",
                           "reef fish", "macroalgae")
         )
  ) %>%
  filter(delta_t < 30)
```

Now produce the plot:

```
deltaT_v_t <- ggplot(cross_source_dat,
                     aes(x = iap_t_mean, y = delta_t, colour = fgroup)) +
  geom_point() +
  geom_smooth(se = FALSE, method = lm, formula = y ~ poly(x, 2)) +
  facet_wrap(~fgroup2) +
  xlab(expression(T[affinity(IAP)])) +
  ylab(expression(paste(T[max], -T[affinity(IAP)]))) +
  theme(legend.position = "none")

ggsave("manuscript/figures/figure 3.png", deltaT_v_t,
       width = 12, height = 8, units = "in")
```

This is Figure 3 in the ms, showing the difference between temperature affinity and Tmax across benthos, fish, and macrophytes, as a function of IAP T-at-depth temperature affinity. These figures combine Tmax data from both the Comte-Olden and GlobTherm databases. Fish are further divided into demersal (khaki), benthopelagic (green), pelagic (light blue) and reef-associated (dark blue) species. Lines are fits from second order polynomial models.
